# Enhanced Volatile Organic Compounds emissions and organic aerosol mass increase the oligomer content of atmospheric aerosols

**DOI:** 10.1038/srep35038

**Published:** 2016-10-13

**Authors:** Ivan Kourtchev, Chiara Giorio, Antti Manninen, Eoin Wilson, Brendan Mahon, Juho Aalto, Maija Kajos, Dean Venables, Taina Ruuskanen, Janne Levula, Matti Loponen, Sarah Connors, Neil Harris, Defeng Zhao, Astrid Kiendler-Scharr, Thomas Mentel, Yinon Rudich, Mattias Hallquist, Jean-Francois Doussin, Willy Maenhaut, Jaana Bäck, Tuukka Petäjä, John Wenger, Markku Kulmala, Markus Kalberer

**Affiliations:** 1 Department of Chemistry, University of Cambridge, Cambridge, CB2 1EW, UK; 2 Department of Chemistry and Environmental Research Institute, University College Cork, Cork, Ireland; 3 Department of Physics, University of Helsinki, P.O. Box 64, 00014, University of Helsinki, Helsinki, Finland; 4 Department of Forest Sciences, P.O. Box 27, FI-00014 University of Helsinki, Finland; 5 Hyytiälä Forestry Field Station, Hyytiäläntie 124, Korkeakoski, 35500, Finland; 6 Leibniz Institute for Tropospheric Research (TROPOS), Permoserstr. 15, 04318 Leipzig, Germany; 7 Centre for Atmospheric Informatics and Emissions Technology, Cranfield University, Cranfield MK43 0AL, UK; 8 Institut für Energie- und Klimaforschung (IEK-8), Forschungszentrum Jülich GmbH; 9 Department of Earth and Planetary Sciences and Energy Research, Weizmann Institute, Rehovot 76100, Israel; 10 Department of Chemistry, Atmospheric Science, University of Gothenburg, 412 96 Göteborg, Sweden; 11 LISA, CNRS UMR 7583, Universités Paris-Est-Créteil et Paris-Diderot, Institut Pierre Simon Laplace, 61 Avenue du Général de Gaulle, 94010, Créteil, France; 12 Department of Analytical Chemistry, Ghent University, Krijgslaan 281, S12, 9000 Ghent, Belgium; 13 Department of Pharmaceutical Sciences, University of Antwerp, Universiteitsplein 1, 2610 Antwerp, Belgium

## Abstract

Secondary organic aerosol (SOA) accounts for a dominant fraction of the submicron atmospheric particle mass, but knowledge of the formation, composition and climate effects of SOA is incomplete and limits our understanding of overall aerosol effects in the atmosphere. Organic oligomers were discovered as dominant components in SOA over a decade ago in laboratory experiments and have since been proposed to play a dominant role in many aerosol processes. However, it remains unclear whether oligomers are relevant under ambient atmospheric conditions because they are often not clearly observed in field samples. Here we resolve this long-standing discrepancy by showing that elevated SOA mass is one of the key drivers of oligomer formation in the ambient atmosphere and laboratory experiments. We show for the first time that a specific organic compound class in aerosols, oligomers, is strongly correlated with cloud condensation nuclei (CCN) activities of SOA particles. These findings might have important implications for future climate scenarios where increased temperatures cause higher biogenic volatile organic compound (VOC) emissions, which in turn lead to higher SOA mass formation and significant changes in SOA composition. Such processes would need to be considered in climate models for a realistic representation of future aerosol-climate-biosphere feedbacks.

Aerosol particles play a vital role in the climate system by scattering and absorbing solar radiation and influencing cloud formation^1^,^2^. Secondary Organic Aerosol (SOA), a significant component of the ambient aerosol^3^,^4^,^5^, is produced from gas-to-particle conversion processes of volatile organic compounds (VOCs) released into the atmosphere from anthropogenic and biogenic sources, with emissions from the latter dominating on a global scale. Numerous laboratory experiments have identified organic oligomers as key components of SOA and it has been proposed that oligomers affect and explain a wide range of important climate-relevant aerosol properties, e.g. the unresolved and highly oxidized organic aerosol fraction, light absorption properties, particle nucleation, particle viscosity or CCN activity e.g. refs 6, 7, 8. Oligomers observed in laboratory-generated SOA show clear chain length distributions with distinct dimers, trimers and higher oligomers^9^, their chemical composition, however, remains largely unknown^10^. This is in stark contrast to observations of ambient aerosols where such oligomer patterns are not clearly observed. In ambient aerosols the number of high-molecular mass compounds is extremely variable and oligomers are often not observed at all, or observed in very small numbers^11^,^12^,^13^. This discrepancy highlights that SOA generated in laboratory experiments is potentially very different in its chemical composition and climate effects from ambient particles.

It is unclear which factors control oligomer formation and whether they affect climate-relevant aerosol properties. To address these questions, we analysed the detailed molecular organic composition of biogenic SOA (BSOA) generated in laboratory experiments and of aerosol collected at a remote boreal forest site^14^ (Hyytiälä, Finland) during two summer periods in 2011 and 2014 representing the largest record of oligomer measurements to date.

## Results and Discussions

**Figure 1:**
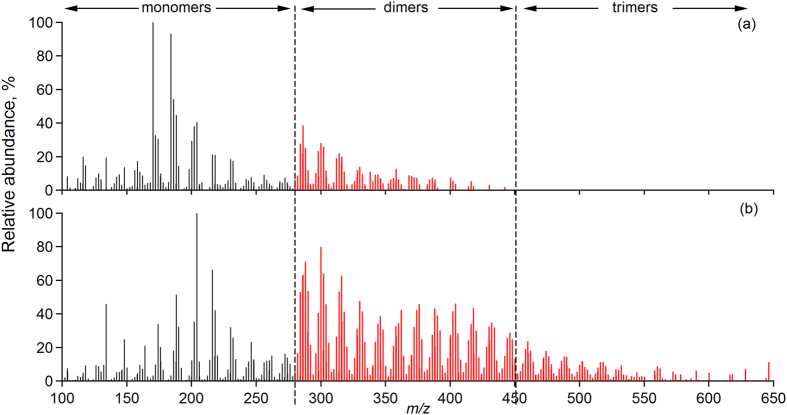
Mass spectra of secondary organic aerosol (SOA) collected at the remote boreal forest site in Hyytiälä, Finland. (**a**) SOA from samples collected in summer 2011 show only a small number of components in the oligomeric mass region (ions above *m*/*z* 280) and no components above *m*/*z* 450 where terpene-trimers are observed (see Fig. 2a–c). (**b**) SOA from samples collected in summer 2014 with clear features in the oligomeric, high molecular mass range up to *m*/*z* 600. Red lines correspond to ions in the oligomeric region which intensities have been multiplied by a factor of 5.

The high-molecular weight, oligomeric part of the mass spectra for the ambient samples is strikingly different in 2011 and 2014 (Fig. 1). BSOA components with masses above *m*/*z* 280 are usually considered as terpene-dimers and compounds above *m*/*z* 450 as trimers^15^,^16^. In the 2011 SOA mass spectrum (Fig. 1a), the overall signal intensity drops significantly above *m*/*z* 320 and almost no peaks are observed above *m*/*z* 400. This is in strong contrast to the 2014 SOA mass spectrum (Fig. 1b), where a large number of ions are observed up to *m*/*z* 600 at significant intensities, indicating an increased number of dimers and the presence of higher oligomers.

**Figure 2:**
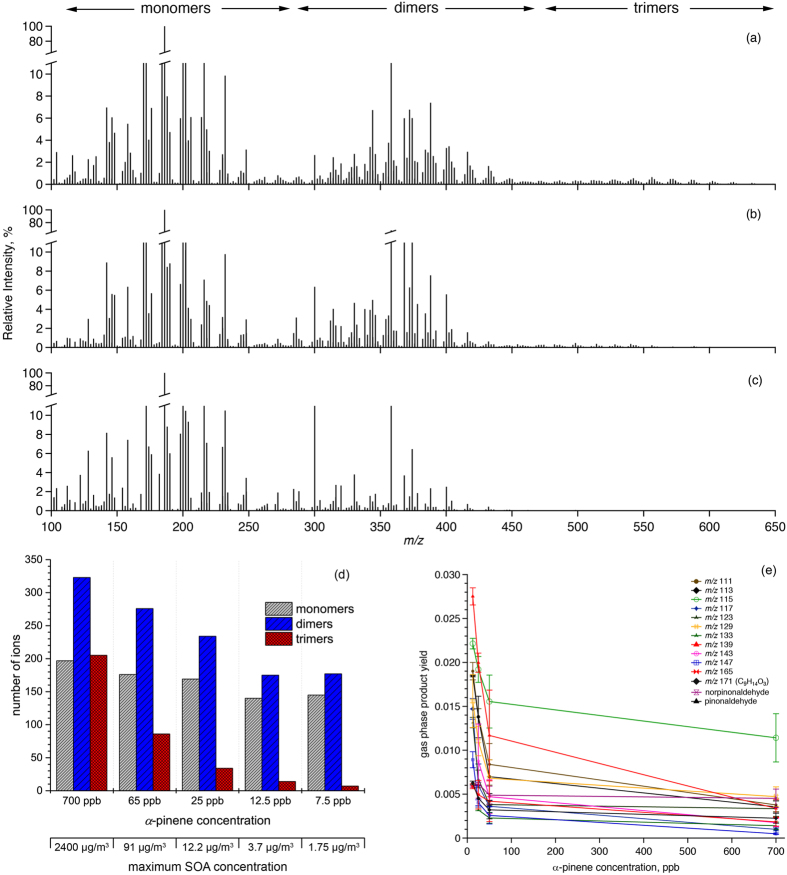
Mass spectra of SOA generated from *α*-pinene ozonolysis in an atmospheric simulation chamber with initial *α*-pinene concentrations of (**a**) 700 ppb, (**b**) 65 ppb and (**c**) 7.5 ppb. Note that relative intensity scales are shown up to 10%. Several monomers and a few dimers have intensities >10%. (**d**) The number of monomers stays relatively constant over all *α*-pinene and SOA mass concentrations (150 to 200 ions) while the number of peaks in the dimer mass region decreases by almost a factor of two and trimers decrease by more than a factor of ten and are essentially not formed under conditions relevant for the ambient atmosphere (7.5 ppb). (**e**) Gas phase product yields (defined as concentration of oxidation product divided by the starting concentration of *α*-pinene) for seven volatile oxidation products of *α*-pinene. Yields decrease with increasing *α*-pinene starting concentration indicating that the species partition increasingly into the particle phase with higher SOA concentrations.

To identify atmospheric conditions that promote or hinder the formation of oligomers we performed atmospheric simulation chamber experiments where *α*-pinene, the most important BVOC in Hyytiälä^17^,^18^ was oxidized via reaction with ozone. The mass spectra in Fig. 2a–d show the composition of SOA generated from a series of ozonolysis experiments with different *α*-pinene mixing ratios between 7.5 ppb and 700 ppb. Experiments with the lowest two *α*-pinene mixing ratios (i.e. 7.5 and 12.5 ppb) reflect the highest monoterpene concentrations observed in Hyytiälä (see Supplementary Fig. S1a), whereas those with higher concentrations (low tens to several hundred ppb) are often used in simulation chamber experiments, especially when detailed chemical characterizations are performed^15^,^19^. The maximum SOA mass generated in the chamber experiments spans three orders of magnitude, ranging from ca. 1.75 μg m^−3^ at 7.5 ppb *α*-pinene to about 2400 μg m^−3^ for 700 ppb *α*-pinene (Fig. 2d and Supplementary Table S1).

The number of ions in the oligomer mass range drops significantly when *α*-pinene and SOA mass concentrations decrease, from about 520 peaks at 700 ppb to about 180 at 7.5 ppb. Trimers disappear almost entirely at the lowest VOC mixing ratios (Fig. 2d). These results clearly demonstrate that SOA mass and precursor concentration govern the formation of oligomers, to the extent that oligomer formation is largely suppressed at SOA masses less than about 4 μg m^−3^ and at terpene levels below ca. 10–15 ppb. The smaller number of dimers and the virtual absence of trimers (i.e. > *m*/*z* 450) in the 1.75 μg m^−3^ experiments closely resembles the BSOA composition in the ambient atmosphere from 2011 (Fig. 1a).

An examination of the organic gas phase composition in chamber experiments reveals that many gaseous BVOC oxidation products increasingly partition from the gas phase into the particle phase at higher SOA concentrations. Fig. 2e shows that gas phase yields of 14 representative *α*-pinene oxidation products decrease by a factor of more than 2 with increasing SOA concentrations (see also Supplementary Fig. S2) most likely due to the increased gas/particle partitioning at high SOA concentrations. This suggests that some of these semi-volatile compounds are involved in oligomer formation and could explain why oligomers are not formed at low SOA mass where these compounds are found predominantly in the gas phase rather than the particle phase.

We performed a wide range of additional chamber experiments to explore the influence of other atmospheric parameters on oligomer formation in BSOA, including (i) long-term atmospheric aging (up to 48 h) under natural sunlight conditions; (ii) the generation of SOA from a mixture of the four most abundant BVOCs present in Hyytiälä representing 80% of biogenic VOCs in Hyytiälä, rather than *α*-pinene only; (iii) the effect of different oxidising conditions, i.e. ozonolysis vs. OH-oxidation/photolysis (Supplementary Fig. S3a–f). None of these conditions prevent oligomer formation significantly or cause their decomposition indicating that once oligomers are formed they are relatively stable under ambient conditions and that VOC precursor and SOA mass concentrations are indeed the dominant factors controlling oligomer formation in aerosols. In our previous work^20^ we demonstrated that SOA formed during ozonolysis of a BVOC mixture under dry conditions (RH < 10%) contained a significant number of oligomers at intensities which are comparable to those observed in the present smog chamber studies performed at RH~55%. Other studies have observed an increase in oligomer formation when comparing completely dry with 40–50% RH conditions^21^,^22^. If RH was a strong driver of oligomer formation in our field samples, we would expect to see more oligomers during the 2011 sampling period when the median RH was 84% compared to 2014 (median RH = 69%). However, the opposite trend was observed. In addition, other authors have shown that acidic seed particles increase oligomer formation but no seed particles have been identified that prevent oligomer formation *e.g*. ref. 23.

To investigate whether the clear dependence of oligomer formation on BVOC and SOA mass concentration in the chamber experiments is also observed in the field and to determine potential climate effects of changes in oligomer content, we examined CCN activities, BVOC and sub-micron particulate matter (PM_1_) concentrations in Hyytiälä during the aerosol sampling periods in 2011 and 2014. The amounts of monoterpenes and isoprene in 2014 (when larger numbers of oligomers were observed) were about 2.5 and 5 times higher, respectively, than during the sampling period in 2011 (see Supplementary Fig. S1). The higher BVOC concentrations in 2014 are most likely caused by the higher day-time temperatures, which were on average 8 °C higher in 2014 (peaking at 29 °C) than in 2011 (see Fig. S4a,b) and which strongly affect temperature-dependent BVOC emissions from trees^24^. Similarly, PM_1_ and organic carbon (OC) concentrations in PM_1_ were almost a factor of 2 higher in 2014 (median concentration 6.5 ± 2.8 μg m^−3^ for PM_1_ and 3.6 ± 0.9 μg m^−3^ for OC) than in 2011 (3.8 ± 2.6 μg m^−3^ for PM_1_ and 2.5 ± 0.26 μg m^−3^ for OC) (see Supplementary Fig. S4e,f).

Thus, the very different atmospheric conditions in 2011 and 2014 mirror the trends observed in the chamber experiments: low organic aerosol concentrations (below about 3 μg m^−3^ in the ambient atmosphere and 3–5 μg m^−3^ in chamber experiments) prevent significant oligomer formation. The corresponding BVOC-concentration for oligomer formation in chamber experiments is about 10–15 ppb *α*-pinene. In the ambient atmosphere this concentration seems lower and monoterpene concentrations of 2–8 ppb (as observed in 2014) are already sufficient to promote oligomer formation. The most likely reason for this difference is that other BVOCs also contribute to SOA mass formation in the ambient atmosphere, such as sesquiterpenes, which produce SOA with very high yields^25^,^26^. It is also possible that OH-initiated oxidation of BVOCs may have played a significant role in additional SOA formation in the ambient air.

**Figure 3:**
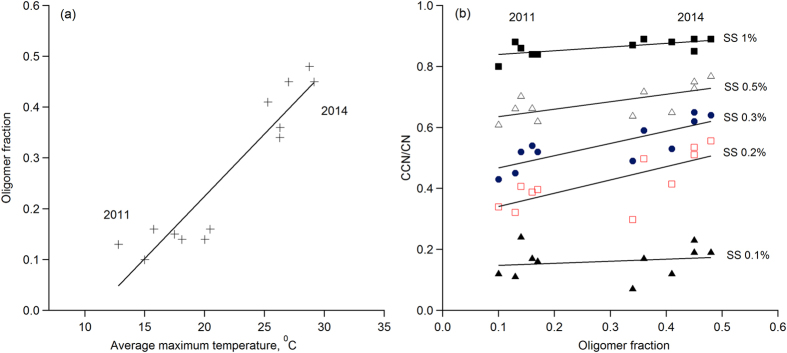
(**a**) Positive relationship between temperature and oligomer fraction in aerosol samples collected at Hyytiälä in summer 2011 and 2014. The oligomer fraction was determined as the average intensities of all oligomer peaks relative to the average intensity of all peaks in the mass spectrum of an individual sample. (**b**) Correlation of CCN/CN with oligomer fraction for all samples in 2011 and 2014 for supersaturations (SS) between 0.1% and 1%. For intermediate SS of 0.2 and 0.3% the CCN/CN ratio increases by up to 30–50%, i.e. from 0.34 to 0.49 for 0.2% SS and 0.47 to 0.61 for 0.3% SS.

This indicates that the oligomer formation observed in Hyytiälä during summer 2014 was driven by higher than average temperatures and BVOC concentrations, which are indicative of a future, warmer climate. Under typical present-day boreal temperatures, however, BVOC and SOA concentrations are too low to promote significant oligomer formation. Figure 3a demonstrates the clear correlation between a higher oligomer fraction in SOA and increasing temperature. Similarly, higher OC content and monoterpene concentrations correlate positively with higher oligomer fractions (Supplementary Fig. S5a–c), supporting the findings of our chamber experiments. Emissions of monoterpenes, which are the major precursors of SOA and its components (e.g., oligomers) in Hyytiälä, increase exponentially with temperature^24^,^27^. Tingey *et al*.^24^ described monoterpene emission rate by the following equation: 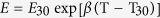 (Equation 1), where *E*_30_ is a standardised emission rate at 30 °C, *β* is an empirical coefficient, T is a leaf temperature and T_30_ is a standardised leaf temperature at 30 °C. Due to this exponential behaviour the estimated changes in monoterpene emission rates are significantly higher (i.e. up to four times, assuming an average *β* value^27^ of 0.09) for the temperature difference measured *between* the 2011 and 2014 sampling periods, compared to that observed *within* the 2011 period (as shown in Fig. 3a). It must be noted that monoterpene ambient concentrations are not only affected by emission rates but also by loss due to photochemistry and atmospheric dilution.

Higher temperatures are shown to promote SOA evaporation^3^. However, the higher oligomer content of SOA formed at higher temperatures (caused by the higher VOC emissions) will likely lead to less evaporative mass loss due to the significantly smaller vapour pressures of oligomers. In this respect, a recent study by Zhang *et al*.^22^ demonstrated that both temperature and RH may affect the particle phase fraction of a few dimers that were identified using LC/MS. A higher degree of oxidation (see Supplementary Fig. S6) also produces SOA with decreased volatility. These compositional changes will make SOA particles more stable towards evaporation and thus will likely result in a greater direct radiative effect when integrated over the entire atmospheric lifetime of the SOA particle.

**Figure 4:**
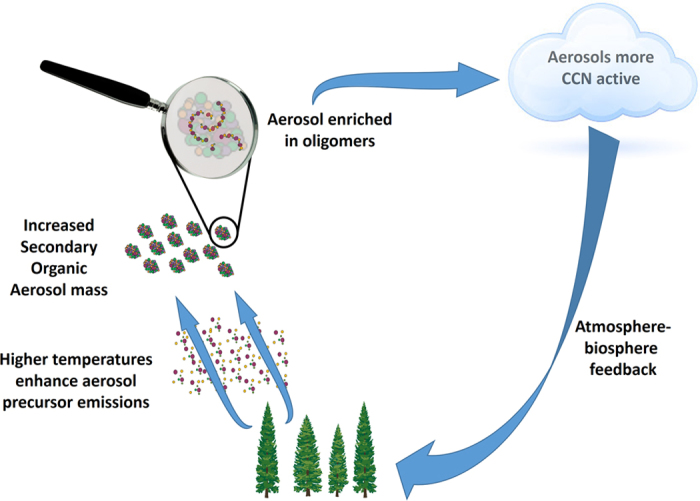
Higher temperatures, likely to occur more frequently in the future, cause higher BVOC emissions and increased SOA concentrations. This leads to higher oligomer content in SOA, which increases their CCN activity. Particles with higher CCN activity will lead to atmosphere-biosphere negative feedbacks as they affect the radiative balance of the atmosphere.

The increased oligomer content of SOA particles showed a strong relationship with another key climate-relevant aerosol property: our field measurements show that the fraction of CCN-active particles (i.e. the CCN/CN ratio) increased in 2014 compared to 2011 by up to 30–50% and correlates with higher oligomer fractions, most notable for 0.2–0.5% supersaturations (Fig. 3b). This correlation could possibly indicate that higher amounts of oligomers are able to speed up the growth of particles to CCN sizes enhancing the CCN/CN ratio. The generally more highly oxidized SOA generated by the higher level of photochemical activity in 2014 (Supplementary Fig. S6d) likely also contributed to the higher CCN/CN ratio in 2014, however, other factors that impact this ratio cannot be entirely excluded and warrant more laboratory and field experiments. The higher temperature in 2014 did not only lead to changes in SOA composition and CCN activity but also increased boundary layer CCN burden (Supplementary Fig. S10, as observed also in ref. 28), which will likely lead to feedbacks^29^ with the biosphere as summarised in Fig. 4.

Although we cannot exclude that other factors e.g., aerosol acidity, oxidant type and possible temperature driven evaporation of monomers from SOA can potentially contribute to the observed differences, our laboratory experiments clearly demonstrated that concentration of VOCs and SOA mass showed a very strong effect on the oligomer formation. We determined atmospheric conditions causing the formation of oligomers in SOA, solving a long-standing apparent discrepancy between laboratory chamber experiments and field measurements. They demonstrate that under the prevailing present-day atmospheric conditions oligomer formation is less relevant for BSOA composition. However, under very warm conditions, such as those encountered in summer 2014 (in Hyytiälä), higher BVOC and SOA concentrations lead to enhanced oligomer formation and increased CCN activity of SOA particles^28^, indicating that in a warmer future climate, oligomers could become an important climate-relevant SOA fraction. This negative feedback might counteract warming effects in the atmosphere and should be included in future climate studies. Future work is needed to better understand whether oligomer component or other coexisting factors influence CCN activity.

## Methods

SOA formation experiments were performed in three different atmospheric simulation chambers at University College Cork (Ireland), University of Cambridge (UK) and Research Center Jülich (Germany) and are detailed in SI.

### Ambient aerosol collection

Aerosol sampling was conducted at the boreal forest site SMEAR II in Hyytiälä, southern Finland (61°51′N, 24°17′E, 181 m above sea level). The forest around the station is dominated by conifers (mainly Scots pine and Norway spruce) with some deciduous trees, such as aspen and birch, and has a tree density of about 2500 ha^−1^. Detailed descriptions of the site, instrumentation, and meteorological data collection are given elsewhere^14^,^30^.

PM_1_ aerosol samples were collected on quartz fiber filters (Pallflex Tissuquartz 2500QAT-UP, 47 mm diameter) and Teflon filters (PALL, Teflon, 47 mm 2.0 μm) using a three-stage Dekati PM_10_ impactor (Dekati Ltd., Tampere, Finland). Before use, the quartz filters were preheated at 600 °C for 12 h to remove organic impurities. The airflow through the sampler was approximately 35 L min^−1^. During the 2011 campaign, ten separate day and night atmospheric aerosol samples were collected (12-hour sampling time for each filter) from 16 August to 25 August, 2011. During the 2014 campaign, eight 48- and 64-hour samples were collected from 7 July to 4 August. In addition, several procedural blanks were collected for 2−3 min. A detailed description of the aerosol sampling was given elsewhere^11^. After collection, the aerosol samples were transferred into Petri dishes and stored at −20 °C until analysis. Meteorological parameters (i.e., temperature, UV-B) and BVOC and PM_1_ concentrations during the sampling periods are shown in Supplementary Figs S1–S4.

### Ambient VOC, aerosol and CCN measurements

Above-canopy isoprene and total monoterpene volume mixing ratios were measured using a Proton Transfer Reaction–Quadrupole Mass Spectrometer (PTR-QMS, Ionicon Analytik GmbH, Innsbruck, Austria) (Supplementary Fig. S1). The same instrument, calibration method, standard gas sampling setup was used in 2011 and 2014, as described in detail elsewhere^31^,^32^,^33^. The length of the heated sampling tube was 100 m in 2011 and 157 m in 2014. The inner diameter of the tubing was 14 mm, the sample flow rate was 43 L min^−1^, and the sample from the sample tube to PTR-QMS was taken through a 5 m long tube with inner diameter of 1.57 mm at a flow rate of 0.1 L min^−1^. The sampling height was 16.8 m. The PTR QMS was calibrated 2–3 times per month by measuring the signal of the standard gas, containing 1 ppm *α*-pinene and isoprene, after diluting the concentration close to the atmospheric concentrations with zero air generated using Parker ChromGas model 3501 (Parker Hannifin, Cleveland, USA) zero air generator.

The aerosol number-concentration size distribution has been continuously measured since 1996 with a Differential Mobility Particle Sizer (DMPS) at the SMEAR II site at Hyytiälä, Finland. A typical DMPS setup is described in detail elsewhere^34^. The CCN measurement setup at the SMEAR II site is described in detail elsewhere^35^.

### Identification of SOA components in ambient aerosol samples

The chemical composition of ambient aerosol samples is a signature of the entire atmospheric lifetime of the particles, which is often several days. Thus, even at remote forest sites like Hyytiälä, the organic fraction of aerosol particles may be composed not only of BSOA components but also primary biogenic and anthropogenic components, as well as organics originating from marine environments. As shown in Supplementary Fig. S7a, the combined 72 h back trajectories, calculated using NAME^36^ of the entire 8-day sampling period in 2011 cover a wide area in Northern Europe, including marine areas and regions with strong anthropogenic influence such as Southern Finland, Sweden, the Baltic States, and Russia^11^.

In contrast, back trajectories of filter samples collected over a shorter time period (e.g., 12 to 48 h) often have similar, geographically more consistent source regions (Supplementary Fig. S7b,c) and therefore the sources that might have influenced the composition of each of the aerosol samples show a smaller variability compared to the potential source regions of the entire field campaign shown in Supplementary Fig. S7a. The samples collected over 12–48 h therefore have compositions that represent their sources, which is likely different from one sample to another. All samples have BSOA components as a common feature in their mass spectra as they all picked up BSOA components over the boreal forest, but they differ in other sources (and thus composition) due to their different air mass history.

To extract the BSOA composition from the complex mixture of natural and anthropogenic components present in each sample we defined only those ions as BSOA that were present in all samples collected over the 1–2 week periods in Hyytiälä in 2011 and 2014, respectively (as shown in Fig. 1a,b). Peaks that were present in only one sample or only a few were disregarded in the spectra shown in Fig. 1. While the intensities of monomer ions (i.e. *m*/*z* 100–270) in the mass spectra in Fig. 1a,b differ, the number of ions and their chemical composition present in the samples from the two years were not substantially different (as confirmed by having 85% of common ions during both periods,172 ions out of 203 ions in the monomeric region).

In addition, as discussed for Fig. 2e, different SOA masses affect gas/particle partitioning of many compounds, leading to changes in their relative abundance in the two samples. The different ion intensities of monomers in Fig. 1a,b could also arise from differences in the mixture of VOC precursor concentrations. However, the differences in oligomer content observed in SOA composition as discussed above and shown in Fig. 1 between 2011 and 2014 were so significant that they are also clearly visible in individual samples, especially in the oligomer region of the mass spectrum and not only in the extracted BSOA spectra. The mass spectra of two individual samples in 2011 and 2014 are shown in Fig. S8.

### High resolution mass spectrometry analysis

Depending on the aerosol loading of the filter samples, which varied between 3 and 150 μg per filter, a part of the quartz fibre filter (5–30 cm^2^) was extracted three times with 5 mL of methanol (Optima TM grade, Fisher Scientific) under ultrasonic agitation in slurry ice for 30 min. The extracts were combined, filtered through a Teflon filter (0.2 μm, ISO-DiscTM Supelco), and reduced by volume to approximately 30–200 μL under a gentle stream of nitrogen. The sample was split into two parts for direct infusion and LC/MS analyses. The concentration of the SOA extracts for direct infusion analysis was adjusted to the same level of approximately 0.25 μg organic carbon μL^−1^. The LC/MS portion was further evaporated to 20 μL and diluted with 0.1% aqueous solution of formic acid to 100 μL.

The final extracts were analysed using a high resolution LTQ Orbitrap Velos mass spectrometer (Thermo Fisher, Bremen, Germany) equipped with electrospray ionization (ESI) and a TriVersa Nanomate robotic nanoflow chip-based ESI (Advion Biosciences, Ithaca NY, USA) sources. The analytical procedure is described in SI.

It is emphasised that the observed decrease in the number of oligomers in samples generated at low *α*-pinene concentrations in the simulation chamber experiments is not due to reaching the detection limit of the mass spectrometer, as all aerosol extract solutions injected into the mass spectrometer were adjusted to about the same concentration of extracted SOA (*ca*. 0.2 μg μL^−1^). The average overall absolute intensity and number of peaks in the low-mass (monomer) range of the mass spectra remain largely unchanged as the BVOC concentration changes by a factor of 100 from 700 ppb to 7 ppb, supporting the observation that the lack of oligomers at the low *α*-pinene concentrations is not caused by instrumental detection limits. Moreover, in previous studies a number of potential measurement artefacts were investigated, e.g., dilution of samples and in-source fragmentation^11^,^20^ (see also SI) which could potentially influence the formation or suppression of oligomers or alter the monomer to oligomer ratios observed in the mass spectra. None of these factors was found to significantly affect oligomers observed in organic aerosol samples and thus the influence of these measurement artefacts is likely minimal.

No current technique can quantify 100 s of oligomers in organic aerosol. Therefore, the number of different oligomer ions in the mass spectra of an aerosol sample was used to assess their importance because the presence of more oligomer peaks in the mass spectra indicates that the reaction conditions promoting oligomer formation become more favorable and thus the number of different oligomers increases. The observed differences in oligomer content from direct infusion analyses are consistent with and supported by quantitative LC/MS analysis, which show unambiguously (Supplementary Fig. S9s vs. Supplementary Fig. S9b) that oligomer concentrations in 2014 were significantly higher than in 2011.

### Additional atmospheric simulation chamber experiments

In addition to SOA precursor concentrations, a range of other reaction conditions was explored to test whether they would have a significant effect on oligomer formation:

i) The effect of long-term atmospheric aging (with ozone and OH radicals) on oligomers was tested at the large-scale outdoor chamber SAPHIR (under natural sunlight conditions for up to 48 h, a time scale not explored so far for oligomer formation, Supplementary Fig. S3a,b). Oligomer formation with masses up to *m*/*z* >650 was readily observed at BVOC concentrations of 112 ppb. Prolonged aging over two days did not result in a decrease or decomposition of the oligomers. These results clearly demonstrate that the exposure of SOA to natural sunlight over two full days does not cause a decrease or decomposition of oligomers and indicate that these compounds are relatively stable and not readily photolysed under ambient conditions.

ii) BSOA chamber experiments are often performed using only one or two VOC precursors e.g., refs 15,37 and 38. To mimic conditions in Hyytiälä more realistically BSOA was generated from a mixture of three monoterpenes (*α*-pinene, *β*-pinene and Δ_3_-carene representing 80% of all terpenes at Hyytiälä^17^,^18^) and isoprene at concentration ratios measured in Hyytiälä. The oligomer content of BSOA generated from this BVOC mixture was only slightly lower than in SOA generated from *α*-pinene alone (Supplementary Fig. S3c,d). However, one of the most pronounced differences in the oligomer mass region between the *α*-pinene SOA and BVOC-mixture SOA is the dimer at *m*/*z* 357. This is by far the most intense dimer in the BVOC-mixture SOA and might explain why this species is one of only a few dimers observed in ambient atmosphere samples when chromatographic techniques are applied (see below).

iii) The effect of different oxidation regimes on oligomer formation was examined^20^. The composition of BSOA due to the oxidation of *α*-pinene with ozone (in the presence of OH scavengers) was compared to SOA generated due to OH oxidation (Supplementary Fig. S3e,f). Under both oxidation regimes significant numbers of oligomers were formed. However, a very different set of oligomers was observed in SOA formed from OH-initiated reactions, where there was a significant shift towards higher molecular weight and higher oxidized SOA components in the monomer and dimer regions, as observed in carbon oxidation state (OS_c_) plots (Supplementary Fig. S6a). The OS_C_ was introduced in aerosol science by Kroll *et al*.^39^ to describe the composition of a complex mixture of organics undergoing oxidation processes. The carbon oxidation state was calculated for each molecular formula identified in the mass spectra using the following equation:


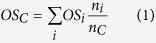


where OS_*i*_ is the oxidation state associated with element *i*, n_*i*_/n_C_ is the molar ratio of element *i* to carbon^39^.

The more highly oxidized OH-initiated SOA closely resembles the degree of oxidation of SOA collected during the warm summer of 2014, suggesting a higher photo-oxidation activity in 2014 (Supplementary Fig. S6b) compared to 2011, when the degree of SOA oxidation more closely resembles ozone-initiated SOA (Supplementary Fig. S6c). The higher photo-oxidation activity can be inferred from the UV-B intensities (relevant for atmospheric photo-oxidation) during 2011 and 2014 with average values 30% higher in 2014 (Supplementary Fig. S4c,d).

## Additional Information

**How to cite this article**: Kourtchev, I. *et al*. Enhanced Volatile Organic Compounds emissions and organic aerosol mass increase the oligomer content of atmospheric aerosols. *Sci. Rep*. **6**, 35038; doi: 10.1038/srep35038 (2016).

## Supplementary Material

Supplementary Information
