# Supplementary material for: Enhanced Volatile Organic Compounds emissions and organic aerosol mass increase the oligomer content of atmospheric aerosols
Source: Sci Rep. 2016 Oct 13;6:35038. doi: 10.1038/srep35038 (PMC5062071; doi:10.1038/srep35038)
Supplement: Supplementary Information [file srep35038-s1.docx]

**Supplementary Information for**

**Enhanced Volatile Organic Compounds emissions and organic aerosol mass increase the oligomer content of atmospheric aerosols**

Ivan Kourtchev^1,2*^, Chiara Giorio^1^, Antti Manninen^3^, Eoin Wilson^2^, Brendan Mahon^1^, Juho Aalto^3,4,5^, Maija Kajos^3^, Dean Venables^2,6^, Taina Ruuskanen^3^, Janne Levula^3,5^, Matti Loponen^3,5^, Sarah Connors^1^, Neil Harris^1,7^, Defeng Zhao^8^, Astrid Kiendler-Scharr^8^, Thomas Mentel^8^, Yinon Rudich^9^, Mattias Hallquist^10^, Jean-Francois Doussin^11^, Willy Maenhaut^12,13^, Jaana Bäck^4^, Tuukka Petäjä^3^, John Wenger^2^, Markku Kulmala^3^, Markus Kalberer^1*^

^1^Department of Chemistry, University of Cambridge, Cambridge, CB2 1EW, UK.

^2^Department of Chemistry and Environmental Research Institute, University College Cork, Cork, Ireland.

^3^Department of Physics, University of Helsinki, P.O. Box 64, 00014, University of Helsinki, Helsinki, Finland.

^4^Department of Forest Sciences, P.O. Box 27, FI-00014 University of Helsinki, Finland.

^5^Hyytiälä Forestry Field Station, Hyytiäläntie 124, Korkeakoski, 35500, Finland.

^6^Leibniz Institute for Tropospheric Research (TROPOS), Permoserstr. 15, 04318 Leipzig, Germany.

^7^Centre for Atmospheric Informatics and Emissions Technology, Cranfield University, Cranfield MK43 0AL, UK.

^8^Institut für Energie- und Klimaforschung (IEK-8), Forschungszentrum Jülich GmbH, 52425 Jülich, Germany.

^9^Department of Earth and Planetary Sciences and Energy Research, Weizmann Institute, Rehovot 76100, Israel.

^10^Department of Chemistry, Atmospheric Science, University of Gothenburg, 412 96 Göteborg, Sweden.

^11^LISA, CNRS UMR 7583, Universités Paris-Est-Créteil et Paris-Diderot, Institut Pierre Simon Laplace, 61 Avenue du Général de Gaulle, 94010, Créteil, France.

^12^Department of Analytical Chemistry, Ghent University, Krijgslaan 281, S12, 9000 Ghent, Belgium.

^13^Department of Pharmaceutical Sciences, University of Antwerp, Universiteitsplein 1, 2610 Antwerp, Belgium.

^*^Correspondence to: i.kourtchev@ucc.ie (I.K.), markus.kalberer@atm.ch.cam.ac.uk (M.K.)

**Atmospheric simulation chamber experiments**

**Chamber experiments in Cork**

Dark ozonolysis experiments with *α*-pinene and a VOC mixture containing *α*-pinene, *β*-pinene, Δ_3_-carene and isoprene were performed in a simulation chamber at the Centre for Research into Atmospheric Chemistry (CRAC), University College Cork^1,2^. The experimental conditions are listed in Supplementary Table S1a. The chamber was a cylinder made of fluorine–ethene–propene (FEP) Teflon® foil with a volume of 3.91 m^3^. It was operated at 296±2 K using purified air at 0.1–1 mbar above atmospheric pressure. The experiments were performed at 55±3% relative humidity (RH) produced by bubbling purified air through heated water. The humidity and temperature were measured using a dew point meter (DRYCAP®DM70 Vaisala). Between experiments the chamber was cleaned by introducing ~1-2 ppmv of ozone into the chamber and flushing with purified air at a flow rate of 0.15 m^3^ min^−1^. Neutral seed particles of ammonium sulfate were generated from 3 mM (NH_4_)_2_SO_4_ (Sigma-Aldrich, 99.99%) solution using an atomiser (TSI® model 3075). BVOCs (i.e., *α*-pinene, *β*-pinene, Δ_3_-carene and isoprene) were introduced into the chamber by flowing purified air over known amounts of the compounds in a gently heated Pyrex impinger. After injecting the BVOCs and allowing them to stabilize for 5-10 min, ozone was introduced into the chamber from an electric discharge generator. Cyclohexane (~40 ppmv, Sigma, >99% purity) was used as an OH scavenger. Particle size distributions (from 19 to 980 nm in diameter) were measured with a TSI® 3080 scanning mobility particle sizer (SMPS) and a TSI® 3010 condensation particle counter. For the SOA mass concentration, the density of the organic material was assumed to be 1.0 g cm^-3^. While this choice of density affects the estimated mass of collected SOA, the major conclusions of this work are not sensitive to the actual value of SOA density. Aerosol samples were collected on pre-baked quartz fiber filters at a flow rate of 30 L min^-1^.

**Chamber experiments in Cambridge**

Dark ozonolysis experiments with *α*-pinene and the same VOC mixture as described above were also performed in the University of Cambridge atmospheric simulation chamber. The experimental conditions are listed in Supplementary Table S1b. The chamber was a collapsible bag made of FEP Teflon® foil with a volume of 5.4 m^3^ operated at 296±2 K. A RH of 55±4% was produced by bubbling purified air through heated water. Between experiments the chamber was cleaned by introducing about ~1-2 ppmv of ozone into the chamber and flushing with purified air. BVOCs, (NH_4_)_2_SO_4_ seed aerosol and an OH scavenger (cyclohexane) were introduced into the chamber in a similar manner as described above for the experiments in Cork. Particle size distributions (from 19 to 980 nm in diameter) were measured with a TSI® 3080 SMPS and a TSI® 3775 condensation particle counter. Aerosol samples were collected on pre-baked quartz fiber filters at a flow rate of 30 L min^-1^.

Online gas phase concentrations of *α*-pinene and its oxidation products were measured using a proton transfer reaction mass spectrometer (PTR-TOF-MS 8000, Ionicon Analytik, Innsbruck, Austria) in the *m/z* range 10-500, with a time resolution of 10 s and a mass resolution m/Δm of 5000 (full width at half maximum) at the mass of protonated acetone. Source settings were: drift tube voltage of 600 V, drift tube pressure at ~ 2.20 mbar, drift tube temperature at 60ºC, resulting in an E/N of ca. 135 Td (1 Td = 10^-17^ V cm^2^). The PTR-TOF-MS inlet (1 m long inert peek tube, 60 ºC, 100 cm^3^ min^-1^ flow rate) was connected to the smog chamber via a Teflon tube (2 m long, Ø 1 mm) kept at room temperature. Data was analysed using the PTR-MS Viewer 3.1 program. The concentration of *α*-pinene was estimated on the basis of the rate constant (k=2.44×10^-9^ cm^3^ molecule^-1^ s^-1^) of the proton transfer reaction^3^ considering both the protonated molecular ion (*m/z* 137.133) and its main fragment (*m/z* 81.070). For the oxidation products of *α*-pinene the rate constant is unknown; therefore, a default rate constant (*k*) of 2×10^-9^ cm^3^ molecule^-1^ s^-1^ was used. The concentration of pinonaldehyde was estimated using both the protonated molecular ion (*m/z* 169.123) and its main fragment (*m/z* 151.112) while for the other oxidation products only the protonated molecular ion was used for calculation^4,5^.

**Chamber experiments in Jülich**

Long duration experiments were performed in the SAPHIR chamber (Simulation of Atmospheric Photochemistry in a large Reaction chamber) at the Research Center Jülich, Germany. SAPHIR is a double-walled Teflon outdoor chamber facility with a volume of 280 m^3^. The chamber uses natural sunlight for illumination and is equipped with a louvre system which can be used to simulate dark processes. The chamber and experimental procedures are described in detail elsewhere^6,7^. In the experiment, a monoterpene mixture of *α*-pinene (48 ppbv) and limonene (48 ppbv) was used and 200 ppbv of O_3_ was added immediately before the roof was opened. The particle number concentration and size distribution were characterised by a condensation particle counter (CPC) and scanning mobility particle counter (SMPS). The chemical composition was characterised using a high resolution time-of-flight aerosol mass spectrometer (HR-ToF-AMS). Aerosol samples were collected on a quartz fiber filter (preheated at 600 °C for 10 h) at a flow rate of 20 L min^-1^ for 1 h with a XAD-4 resin coated annular denuder placed upstream of the filter sampler. The sampling unit was located below the SAPHIR chamber.

**High resolution mass spectrometry analysis**

The Orbitrap MS was calibrated using an Ultramark 1621 solution (Sigma-Aldrich, UK). The mass accuracy of the instrument was routinely checked before the analysis and was below 1 ppm. The instrument mass resolution was 100000 at *m*/*z* 400. A mixture of camphor sulfonic acid (20 ng μL^–1^), glutaric acid (30 ng μL^–1^) and *cis*-pinonic acid (30 ng μL^–1^) in methanol and Ultramark 1621 solution were used to optimize the ion transmission settings.

The direct infusion nanoESI parameters were as follows: the ionization voltage and back pressure were set at −1.4 kV and 0.8 psi, respectively. The inlet temperature was 200 °C. The sample flow rate was approximately 200-300 nL min^-1^. The negative ionization mass spectra were collected in three replicates over ranges *m*/*z* 100–650 and *m*/*z* 150–900 and processed using Xcalibur 2.1 software (Thermo Scientific). As we demonstrated in our previous work^8^ in-source fragmentation tests with a fragmentation voltage of up to 70 V (which is high enough to break non-covalent bonds) showed no significant change in the number of observed oligomers in the mass spectra, thus ruling out the possibility that these oligomers are non-covalent compounds formed in the ESI source. When the fragmentation voltage was increased to 80 V, a decrease in the intensities for both monomers and dimers was observed, confirming that the detected dimers are covalent species.

Liquid chromatography LC/(-)ESI-MS analysis was performed using an Accela system (Thermo Scientific, San Jose, USA) coupled with LTQ Orbitrap Velos MS and a T3 Atlantis C18 column (3 μm; 2.1 × 150 mm; Waters, Milford, USA). The mobile phases consisted of 0.1% formic acid (v/v) (A) and methanol (B). The applied gradient was as follows: 0–3 min 3% B, 3–25 min from 3% to 50% B (linear), 25–43 min from 50% to 90% B (linear), 43–48 min from 90% to 3% B (linear), and kept for 12 min at 3% B (total run time 60 min). MS spectra were collected in full scan using the lock mass at *m*/*z* 91.00368 corresponding to the deprotonated dimer of formic acid with a resolution of 100000 and the mass ranges of *m*/*z* 100–650 and *m*/*z* 150–900. On the basis of pre-scan information from the full scan MS, parallel data-dependent collision induced dissociation (CID) multistage mass spectrometry (MSn) (*n* = 1, 2, 3, and 4) was performed on the most intense precursor ion in three scans at a resolution of 30 000.

**Gas phase reaction yields of *α*-pinene oxidation products**

As shown in Fig. 2e, most masses observed in the PTR-TOF-MS mass spectrum follow a clear trend with lower gas phase yields at higher SOA mass concentration in the simulation chamber. Fig. S2 shows additional masses compared to the selection shown in Fig. 2e.

**Dimer 358 Da, a special dimer**

A relatively small number of dimers, e.g., a dimer with a mass of 358 Da (tentatively identified as pinyl-diaterpenyl ester MW 358 in SOA from ozonolysis of *α*-pinene^9-12^), seems to form also under low BVOC conditions in high enough concentrations to allow characterisation with chromatographic techniques, i.e., LC. Generally, separation and elution of compounds in LC is determined by the column stationary phase thus resulting in the elution of a smaller number of compounds compared to direct infusion mass spectrometry, which was used for the analyses of the samples shown in Fig. 1 and 2. In addition, sample dilution with elution solvent in the LC column often leads to a decreases sensitivity of LC compared to direct infusion. LC-MS analysis can usually separate some tens of compounds whereas with direct infusion MS 1000s of compounds can be separated. In field studies the pinyl-diaterpinyl ester MW 358 has often been reported as the most abundant dimer, typically accounting for about 1% of the total organic aerosol mass. This dimer was also measured in high intensities with LC in the 2014 ambient Hyytiälä samples. In contrast, only traces of the pinyl-diaterpenyl ester MW 358 were found in the 2011 night-time samples but it was not detected in day-time samples (Fig. S9). The dominant appearance of pinyl-diaterpenyl ester MW 358 (compared to other dimers) in field and laboratory samples might be explained with its monomeric building units pinic acid (186 Da) and terpenylic acid (172 Da), which are among the most abundant oxidation products of monoterpenes. Thus, it seems plausible that due to the high concentration of its monomer precursors the pinyl-diaterpenyl ester MW 358 (and a limited number of other dimers, but no higher oligomers) is found in BSOA even at low BVOC concentrations whereas many other oligomers are only formed under higher BVOC concentrations.

**CCN boundary layer burden and CCN/CN fraction**

Higher VOC and SOA mass concentrations and different oxidation regimes not only lead to significant changes in SOA composition as discussed in the main text, but could also explain the differences in cloud condensation nuclei (CCN) properties observed in the field in 2011 and 2014. In addition to changes in CCN/CN discussed in Fig 3b, the total boundary layer burden of CCN number concentration was almost two times higher during the warmer 2014 period (when oligomers were abundant) compared to 2011 (Fig. S10). This can be explained by the higher temperatures in 2014 leading to higher BVOC emissions and PM_1_ concentrations, and supports long-term measurements of aerosol particles concentrations and their BVOC precursors showing that temperature has a very strong effect on the boundary layer burden of CCN^13^.

Besides the oligomer content, other particle properties such as changes in the particle concentration, size distribution or in the inorganic composition could have also influenced the observed changes in the CCN/CN ratio between 2011 and 2014. Larger particles and a higher inorganic particle content would promote an increased CCN/CN ratio.

The aerosol size distribution (Fig. S11, S12) did not change significantly throughout the sampling periods in 2011 and 2014, indicating that the aerosol size distribution was not causing the observed increase in the fraction of CCN-active particles (i.e., the CCN/CN ratio) in 2014. The somewhat higher particle concentrations in 2014 would generally promote a decrease in CCN/CN and therefore cannot explain the higher CCN/CN in 2014.

In addition, sulfuric acid concentrations (calculated using a proxy derived from Chemical Ionization Mass Spectrometry (CIMS) measurements^14^) showed about five times lower values for the sampling period in 2014 compared to that in 2011. Sulfuric acid concentrations are an effective proxy for particle sulfate content and thus the significantly lower values in 2014 cannot explain the observed CCN/CN increase in 2014.

Therefore, changes in particle number, size and inorganic content are likely not affecting significantly the CCN/CN increase observed in 2014, supporting the influence of oligomers in the CCN activity increase in 2014 as discussed in the main text.

References:

1. Kourtchev, I. *et al.* Molecular composition of biogenic secondary organic aerosols using ultrahigh-resolution mass spectrometry: comparing laboratory and field studies. *Atmos. Chem. Phys.* **14**, 2155–2167 (2014).
2. Thüner, L. P., Bardini, P., Rea G. J. & Wenger, J. C. Kinetics of the gas-phase reactions of OH and NO_3_ radicals with dimethylphenols*.* *J. Phys. Chem. A* **108**(50), 11019–11025 (2004).
3. Zhao, J. & Zhang, R. Proton transfer reaction rate constants between hydronium ion (H3O+) and volatile organic compounds. *Atmos. Environ.* **38**(14), 2177-2185 (2004).
4. Lee, A. *et al.* Gas-phase products and secondary aerosol yields from the photooxidation of 16 different terpenes. *J. Geophys. Res. -Atmos.* **111**, D17305, doi:10.1029/2006jd007050 (2006).
5. de Gouw, J. & Warneke, C. Measurements of volatile organic compounds in the earth's atmosphere using proton-transfer-reaction mass spectrometry. *Mass Spectrom. Rev.* **26**(2), 223-257 (2007).
6. Flores, J. M. *et al.* Evolution of the complex refractive index in the UV spectral region in ageing secondary organic aerosol. *Atmos. Chem. Phys.* **14**, 5793-5806 (2014).
7. Zhao, D. F. *et al.* Cloud condensation nuclei activity, droplet growth kinetics and hygroscopicity of biogenic and anthropogenic Secondary Organic Aerosol (SOA). *Atmos. Chem. Phys.* **16**, 1105-1121 (2016).
8. Kourtchev, I. *et al.* Molecular composition of fresh and aged secondary organic aerosol from a mixture of biogenic volatile compounds: a high-resolution mass spectrometry study. *Atmos. Chem. Phys.* **15**, 5683-5695 (2015).
9. Kristensen, K. *et al.* Dimers in *α*-pinene secondary organic aerosol: effect of hydroxyl radical, ozone, relative humidity and aerosol acidity. *Atmos. Chem. Phys.* **14**, 4201-4218 (2014).
10. Camredon, M. *et al.* Distribution of gaseous and particulate organic composition during dark *α*-pinene ozonolysis. *Atmos. Chem. Phys.* **10**, 2893–2917 (2010).
11. Müller, L., Reinnig, M-C., Warnke, J. & Hoffmann, T. Unambiguous identification of esters as oligomers in secondary organic aerosol formed from cyclohexene and cyclohexene/α-pinene ozonolysis. *Atmos. Chem. Phys.* **8**, 1423–143 (2008).
12. Müller, L., Reinnig, M-C., Hayen, H. & Hoffmann, T. Characterization of oligomeric compounds in secondary organic aerosol using liquid chromatography coupled to electrospray ionization Fourier transform ion cyclotron resonance mass spectrometry. *Rapid Commun. Mass Spectrom.* **23**(7), 971–979 (2009).
13. Paasonen, P. *et al.* (2013) Warming-induced increase in aerosol number concentration likely to moderate climate change. *Nat. Geosci.* **6**, 438–442 (2013).
14. Petäjä, T. *et al.* Sulfuric acid and OH concentrations in a boreal forest site. *Atmos. Chem. Phys.* **9**, 7435-7448 (2009).

**Supplementary Information Tables and Figures:**

**Supplementary Table S1a.** Simulation chamber conditions at University College Cork

| Experiment | System | HC*  ppbv | (NH_4_)_2_SO_4_  Seed, µg m^-3^ | SOA**  µg m^-3^ |
| --- | --- | --- | --- | --- |
| 1 | *α*-pinene/O_3_ | 25.5 | 3.3 | 11.1 |
| 2 | *α*-pinene/O_3_ | 25.5 | 2.4 | 10.4 |
| 3 | *α*-pinene/O_3_ | 62.5 | 3.6 | 56.6 |
| 5 | VOC mixture/O_3_ | 1000 | 3 | >2080 |
| 6 | VOC mixture/O_3_ | 120 | 2.7 | 122.6 |
| 7 | VOC mixture/O_3_ | 120 | 4.3 | 157.5 |
| 8 | VOC mixture/O_3_ | 120 | 3.2 | 118.5 |
| 9 | VOC mixture/O_3_ | 62.5 | 2.6 | 40 |
| 10 | VOC mixture/O_3_ | 62.5 | 3.7 | 43 |
| 11 | Chamber blank | - | 3 | <0.1 |
| 12 | Chamber blank | - | 2.7 | <0.3 |

The ozone concentration for all experiments was 3 times the concentration of the hydrocarbons;

* Estimated hydrocarbon (HC) concentrations based on the injected volume of the VOC;

** Maximum SOA concentrations, wall loss and seed corrected.

**Supplementary Table S1b.** Simulation chamber conditions at University of Cambridge

| Experiment | System | HC*  ppbv | (NH_4_)_2_SO_4_  Seed, µg m^-3^ | SOA**  µg m^-3^ |
| --- | --- | --- | --- | --- |
| 1 | *α*-pinene/O_3_ | 700 | 4.3 | >2430 |
| 2 | *α*-pinene/O_3_ | 700 | 3.2 | >2360 |
| 3 | *α*-pinene/O_3_ | 62.5 | 5.8 | 89 |
| 4 | *α*-pinene/O_3_ | 62.5 | 1.8 | 93 |
| 5 | *α*-pinene/O_3_ | 25 | 3.7 | 14 |
| 6 | *α*-pinene/O_3_ | 25 | 1.7 | 10 |
| 7 | *α*-pinene/O_3_ | 12.5 | 3.2 | 4.6 |
| 8 | *α*-pinene/O_3_ | 12.5 | 2.2 | 2.8 |
| 9 | *α*-pinene/O_3_ | 7 | 2.7 | 2.1 |
| 10 | *α*-pinene/O_3_ | 7 | 2.3 | 1.4 |
| 11 | VOC mixture/O_3_ | 700 | 7.1 | >2920 |
| 12 | VOC mixture/O_3_ | 700 | 5.2 | >2640 |
| 13 | VOC mixture/O_3_ | 120 | 3.7 | 122.5 |
| 14 | VOC mixture/O_3_ | 62.5 | 3.9 | 43.5 |
| 15 | VOC mixture/O_3_ | 62.5 | 3.5 | 62.4 |
| 16 | VOC mixture/O_3_ | 31 | 3.8 | 12.4 |
| 17 | VOC mixture/O_3_ | 31 | 4.1 | 11.6 |
| 18 | Chamber blank | - | 3.5 | <0.2 |
| 19 | Chamber blank | - | 3.7 | <0.2 |

The ozone concentration for all experiments was 3 times the concentration of the hydrocarbons;

* Estimated hydrocarbon (HC) concentrations based on the injected volume of the VOC;

** Maximum SOA concentrations, wall loss and seed corrected.


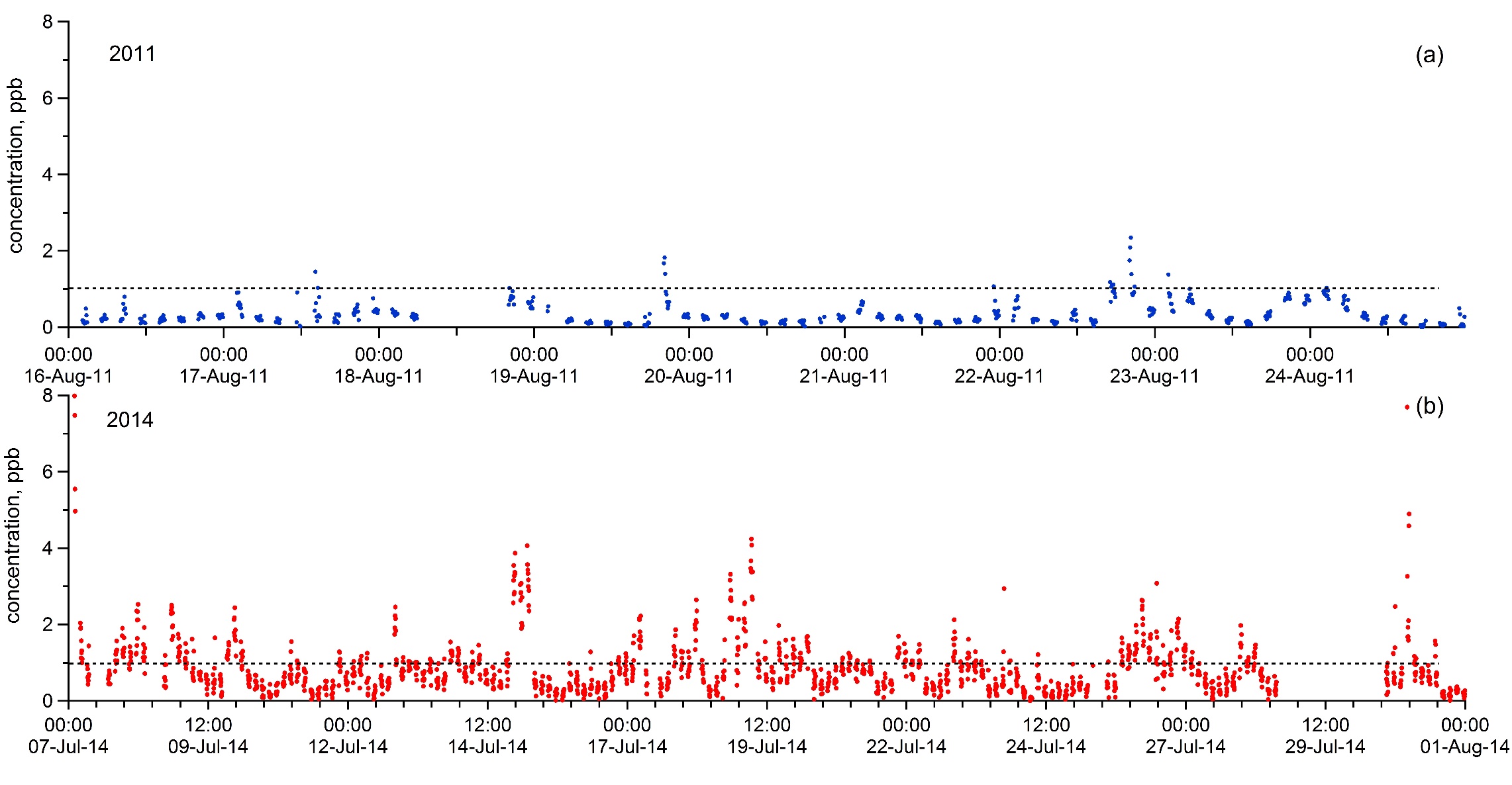


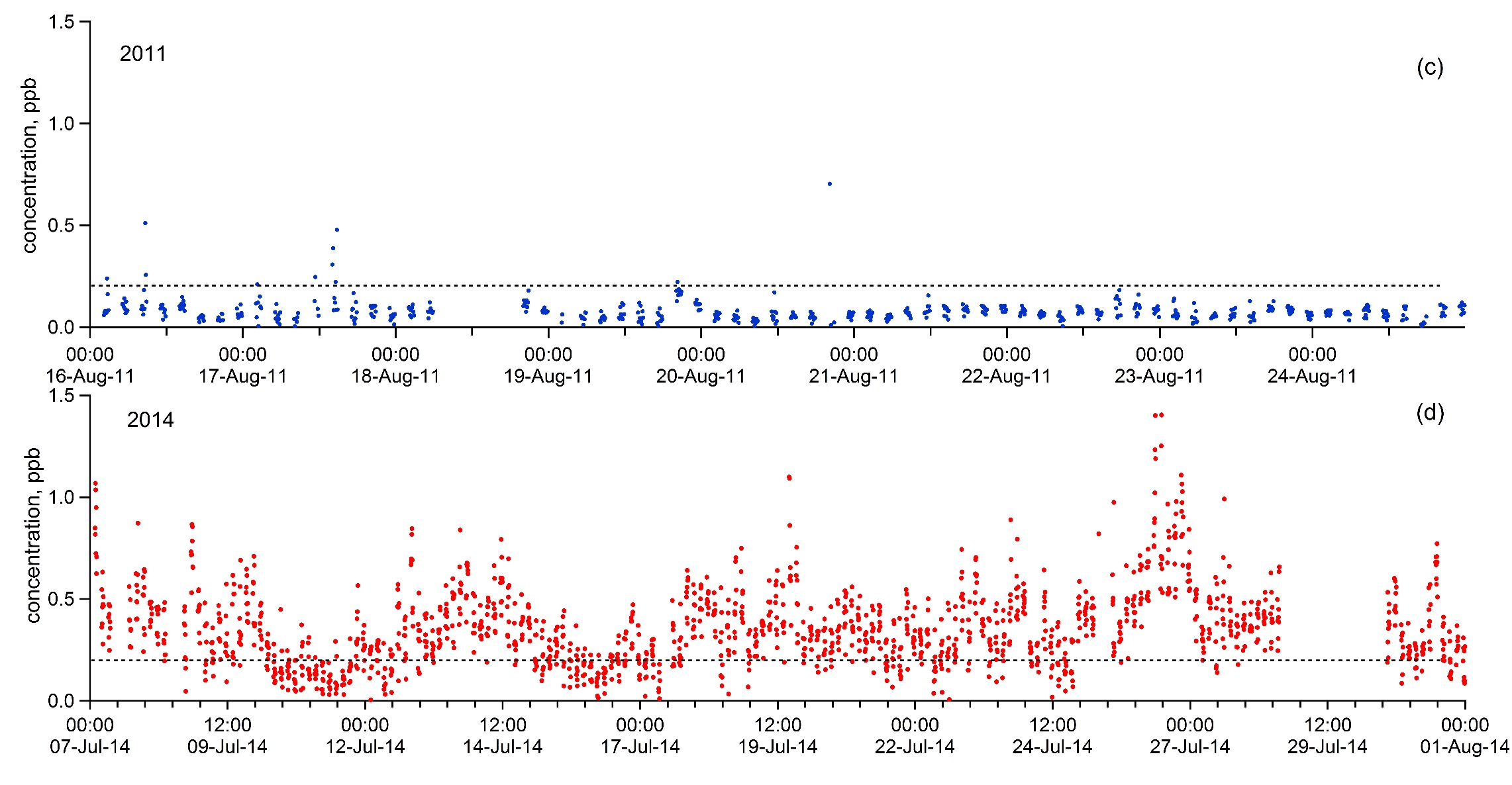


**Supplementary Figure S1.**

**
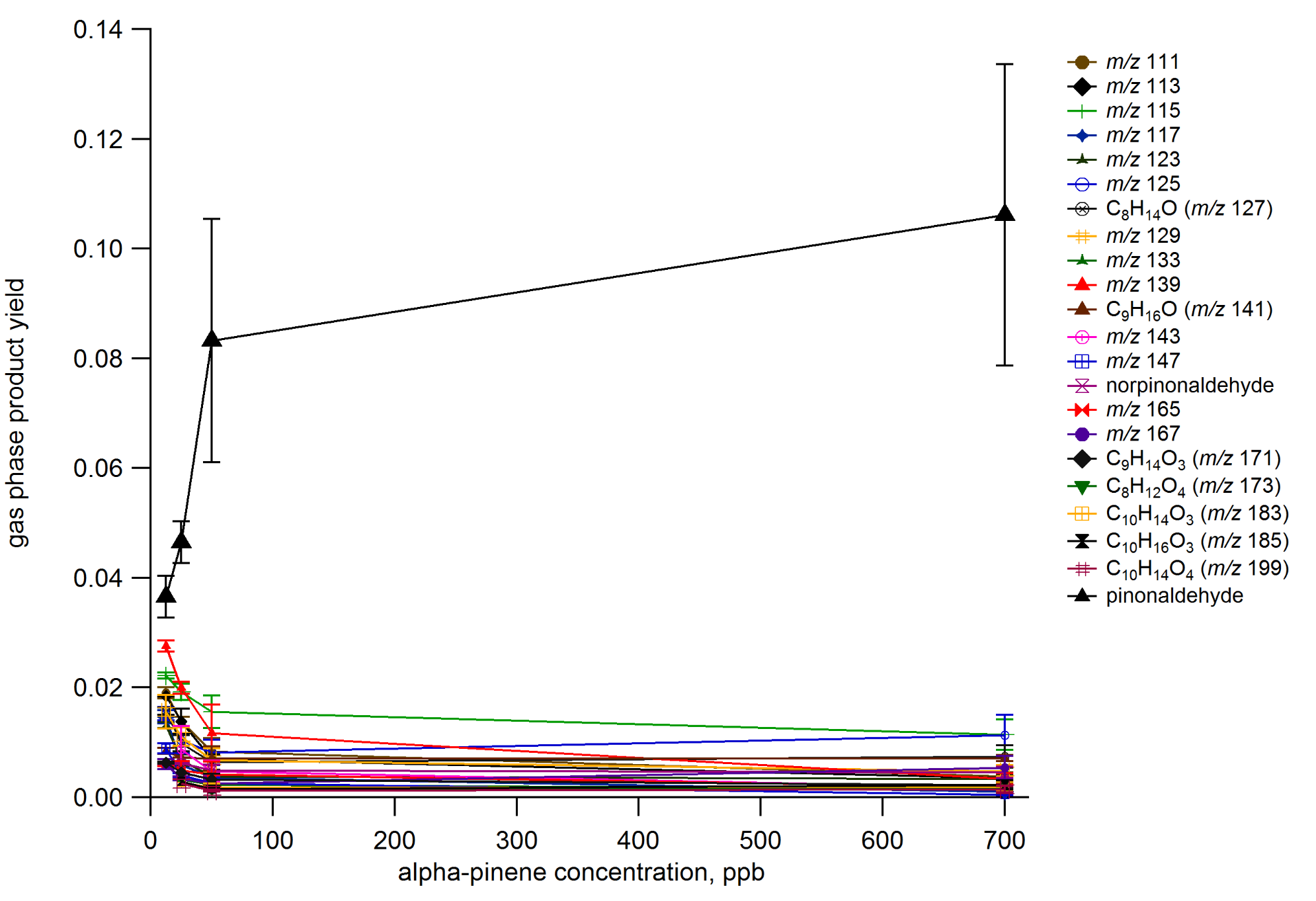
**

**Supplementary Figure S2.**


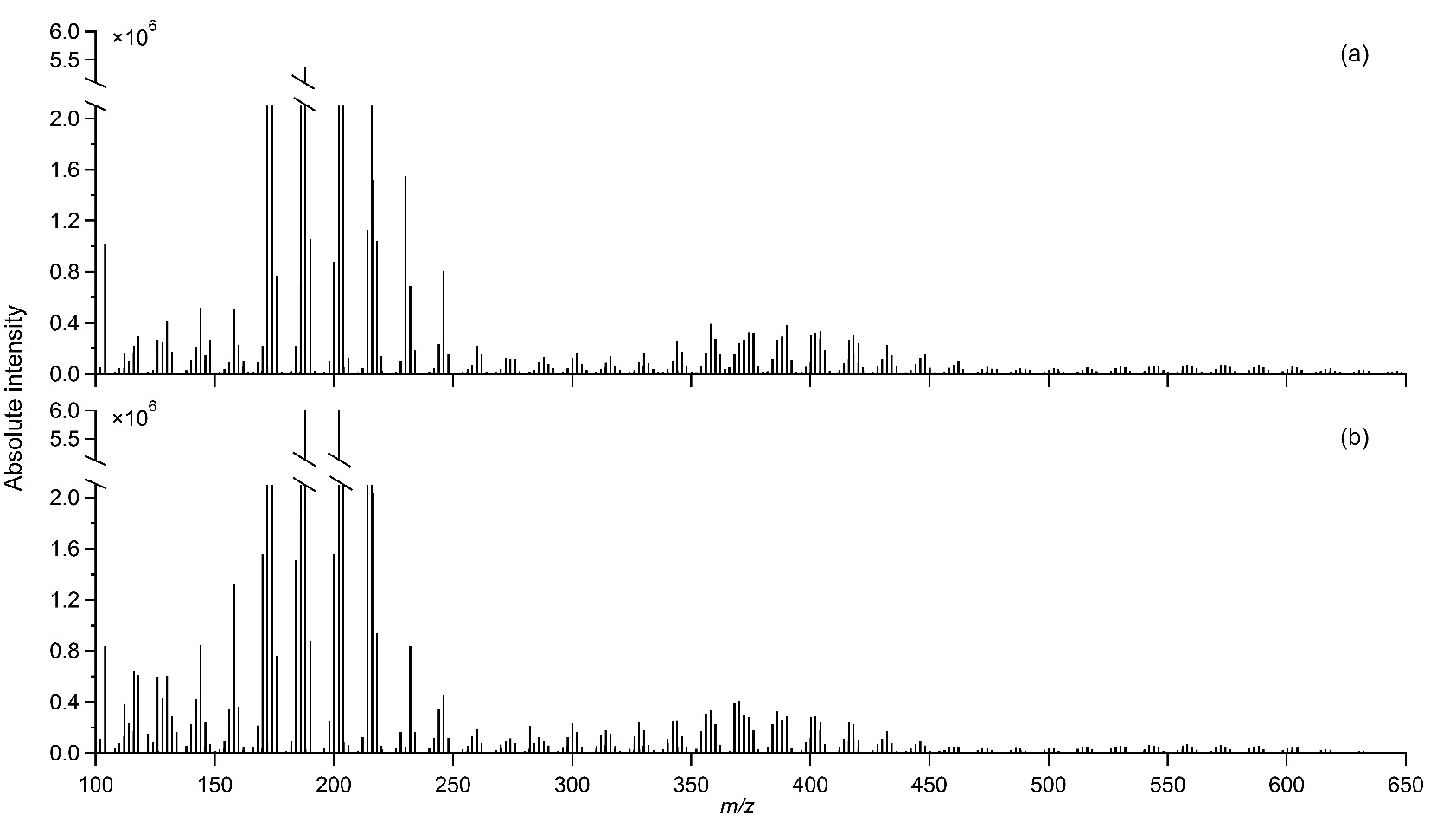


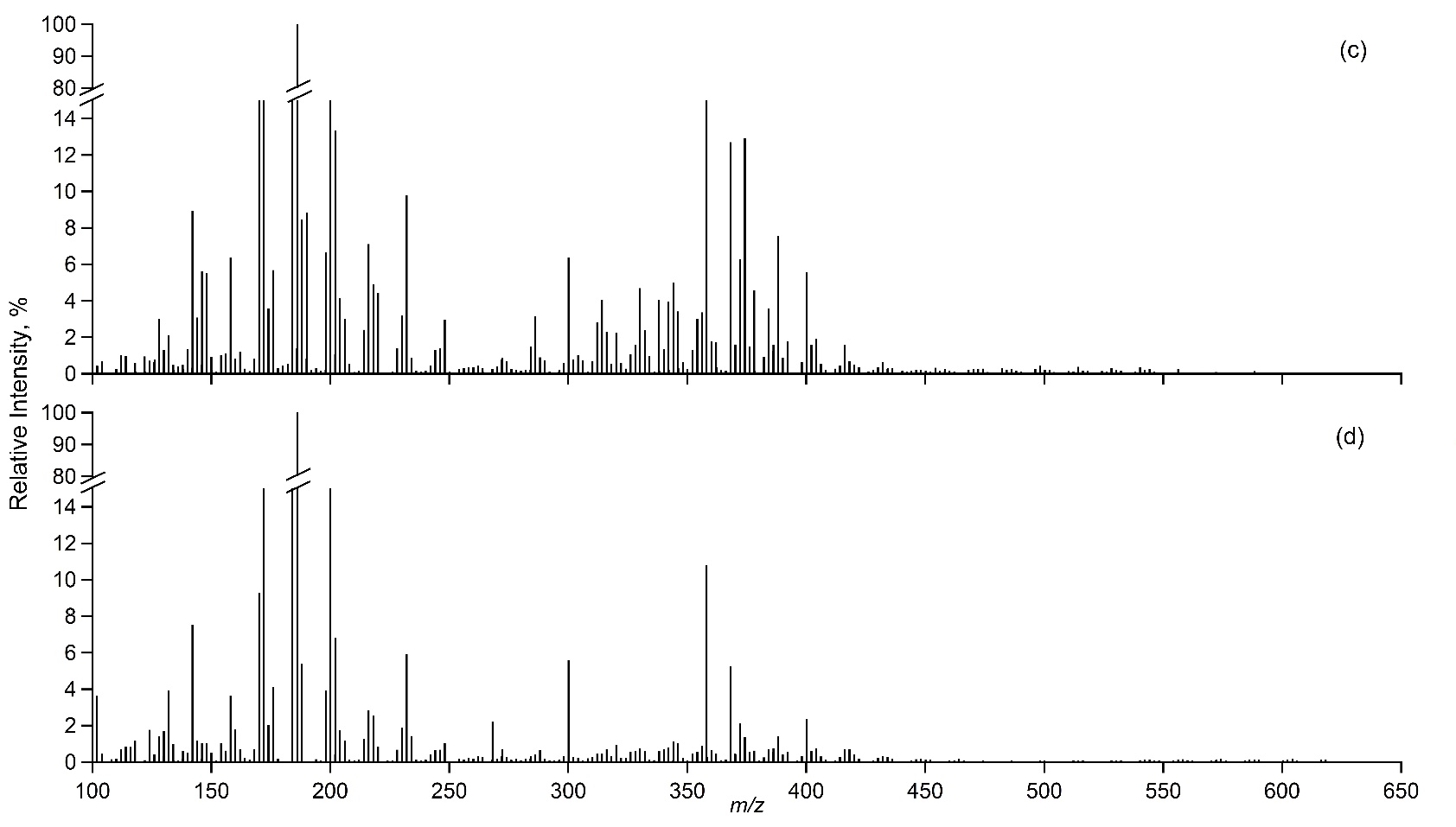


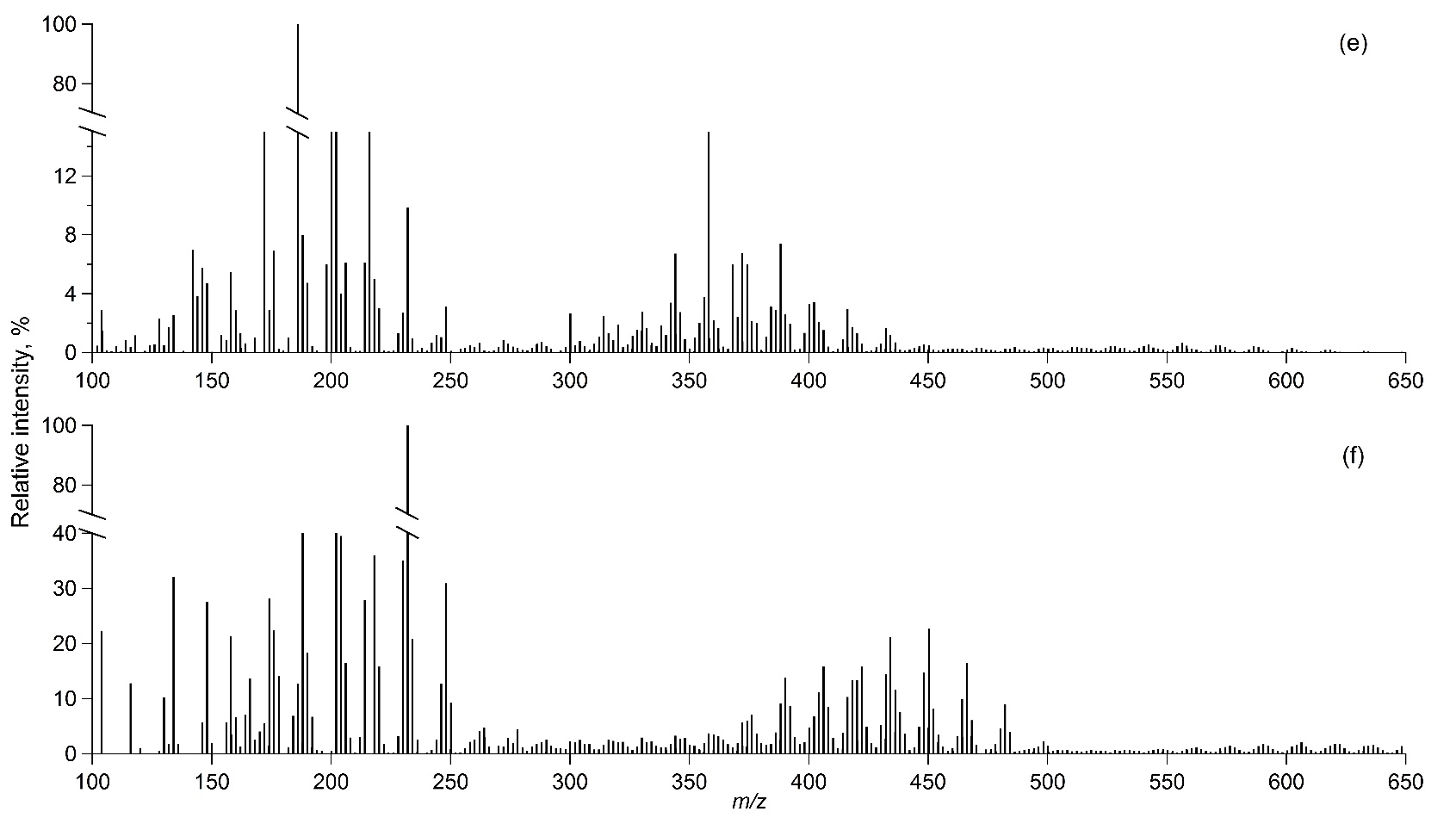


**Supplementary Figure S3.**

**
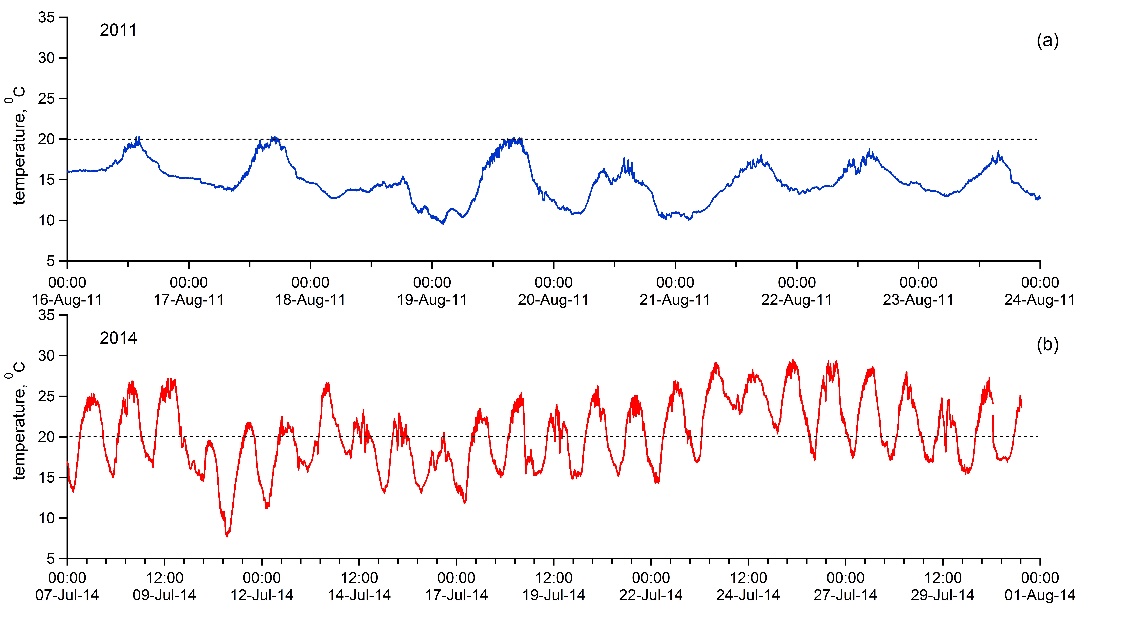
**

**
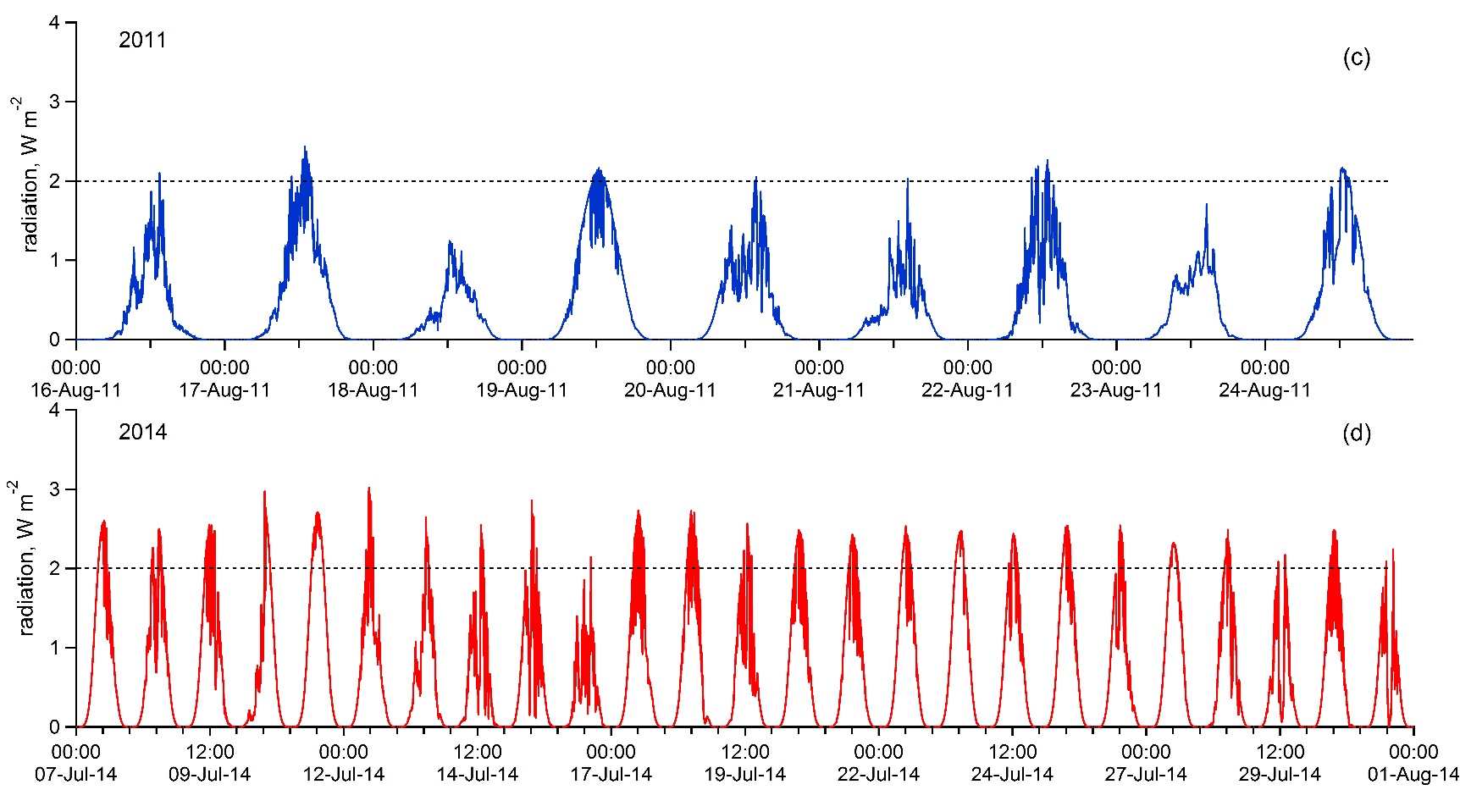
**

**
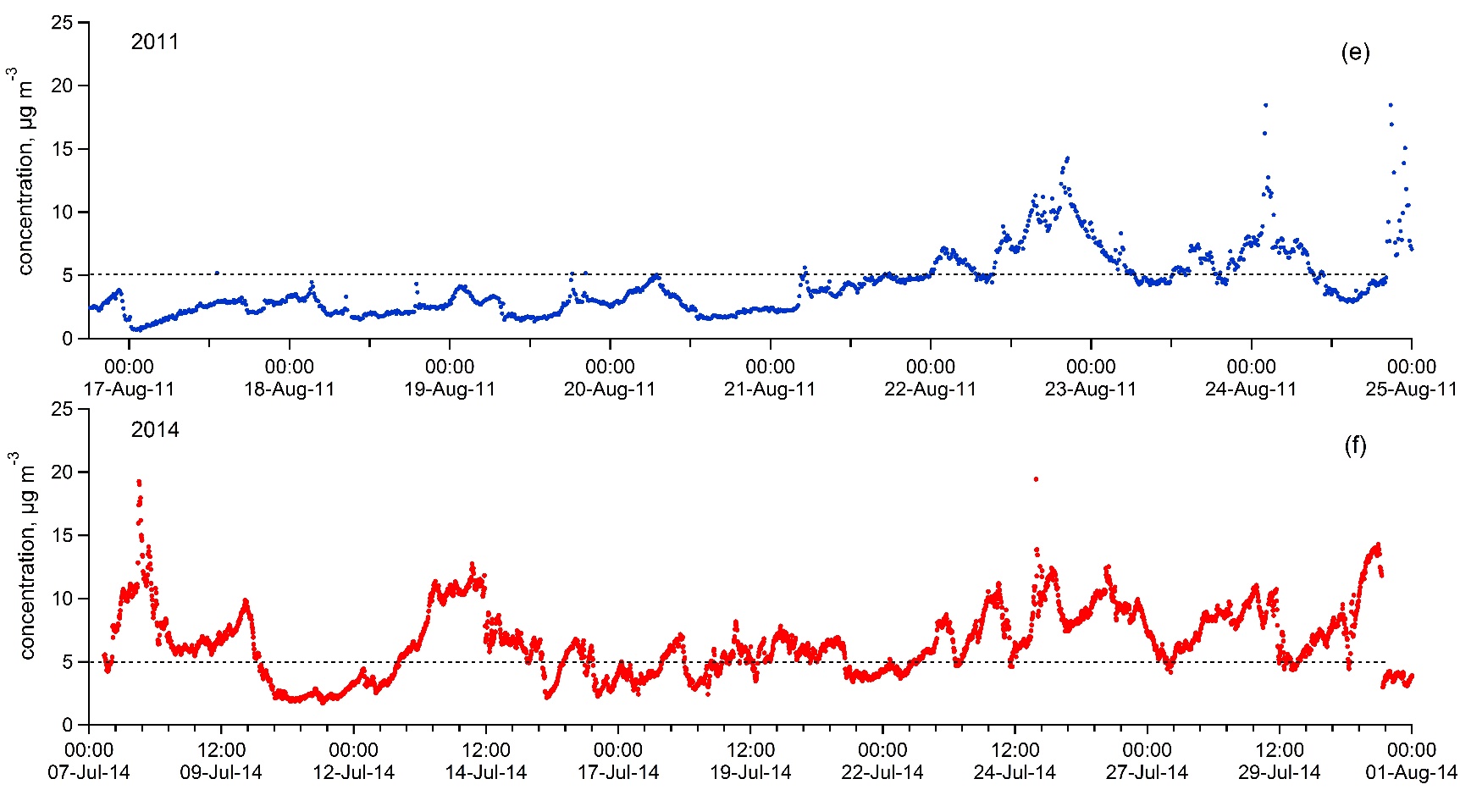
**

**Supplementary Figure S4**.


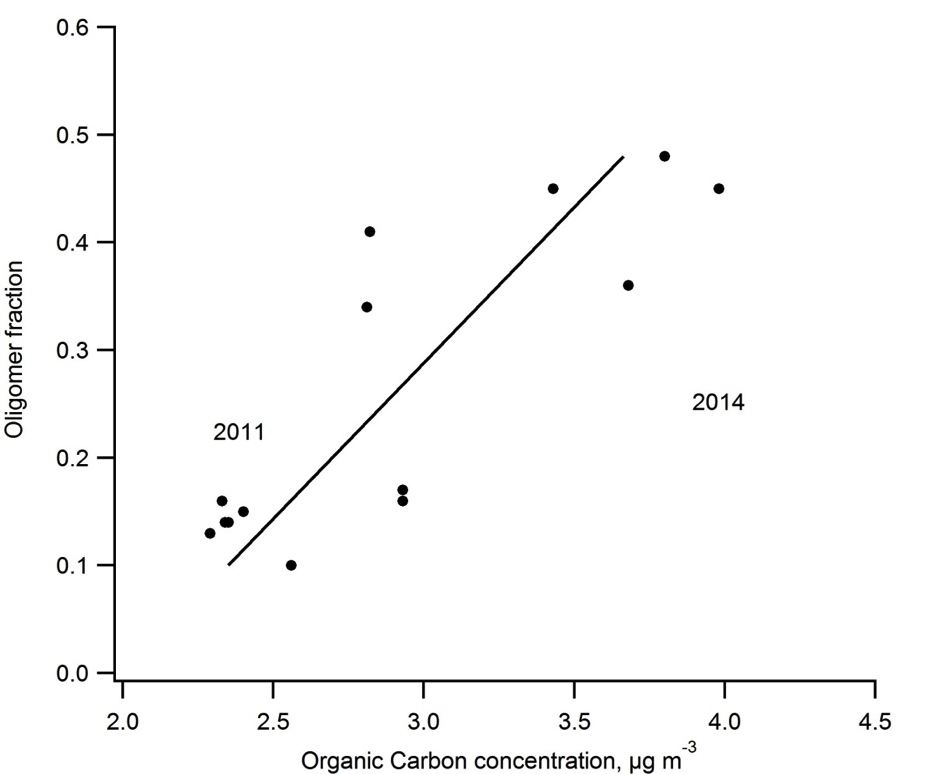


**Supplementary Figure S5a.**


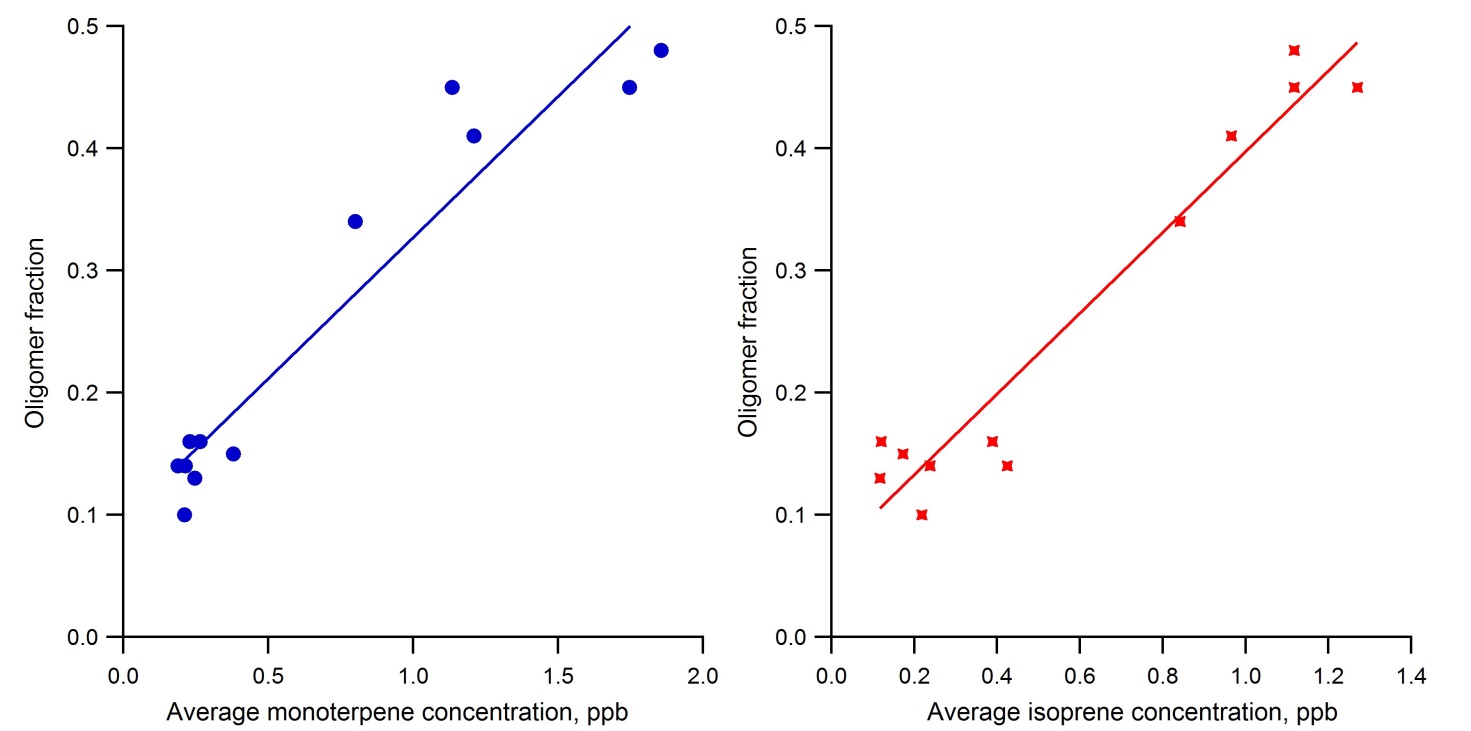


**Supplementary Figure S5b.**

**
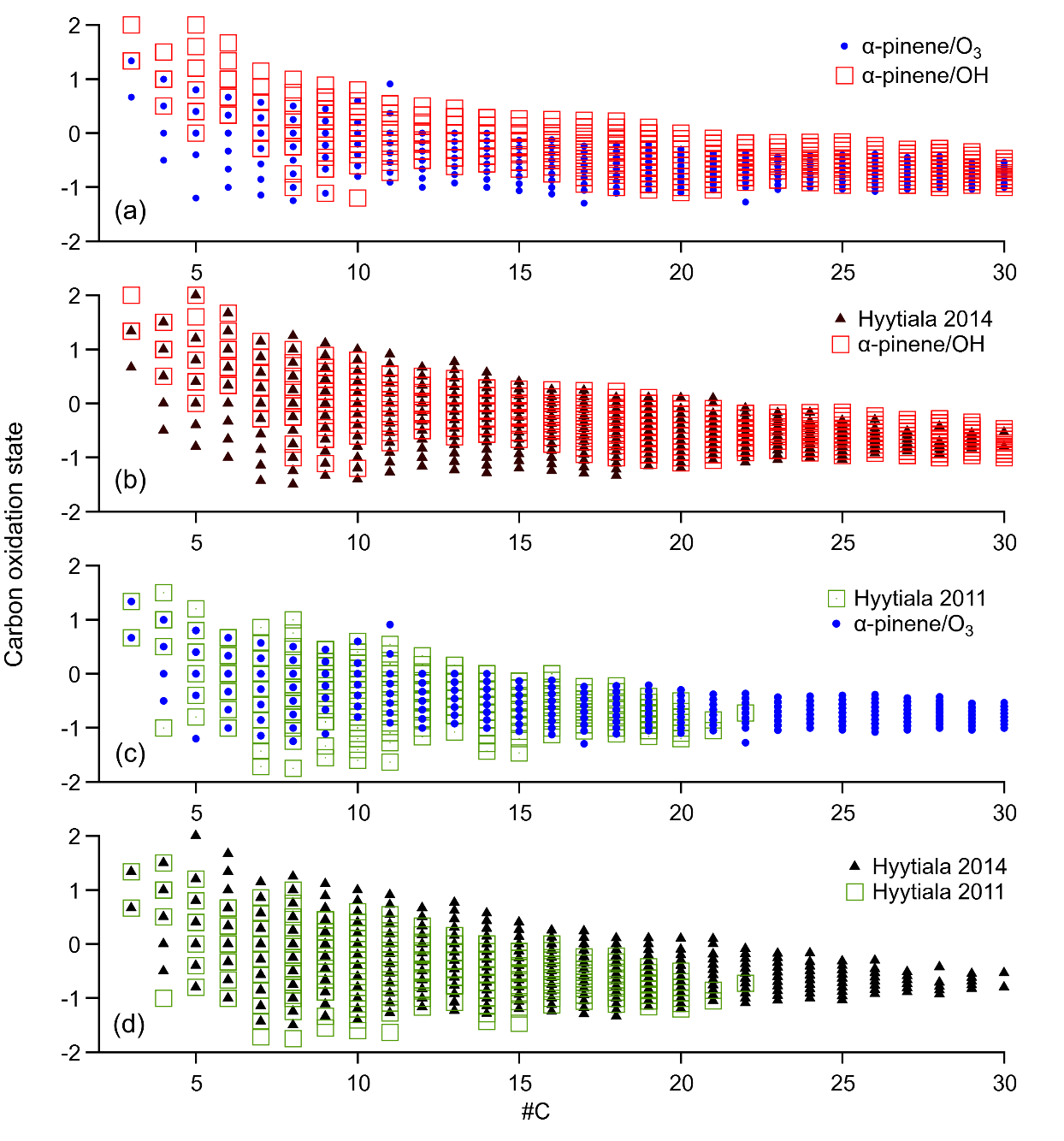
Supplementary Figure S6.**


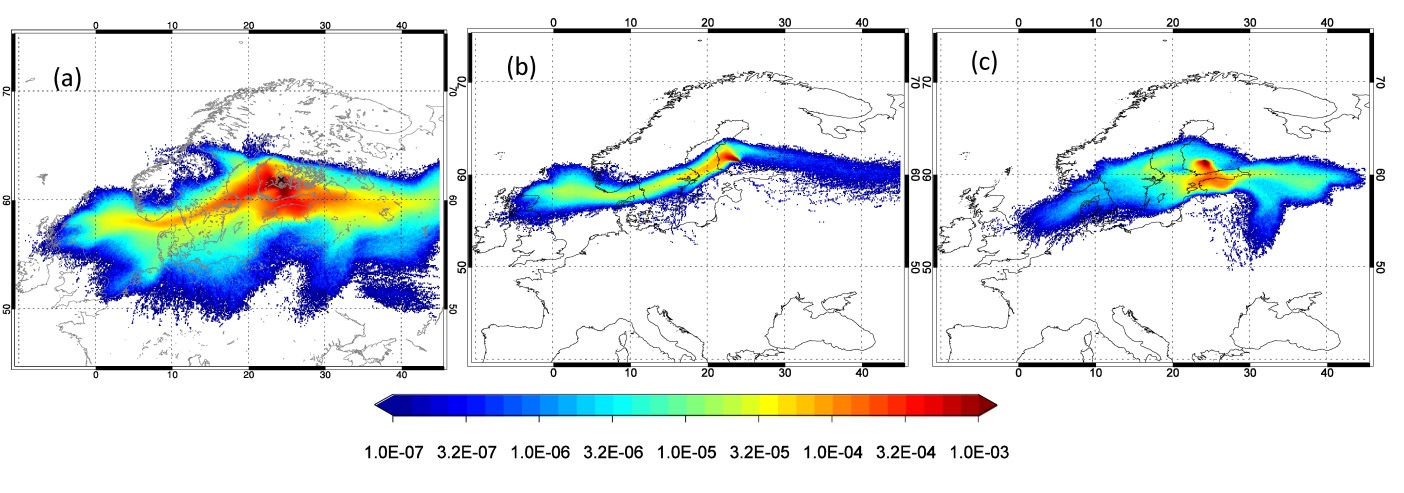


**72-hour integrated particle density 0-100 m (g s m^-3^)**

**Supplementary Figure S7.**

**
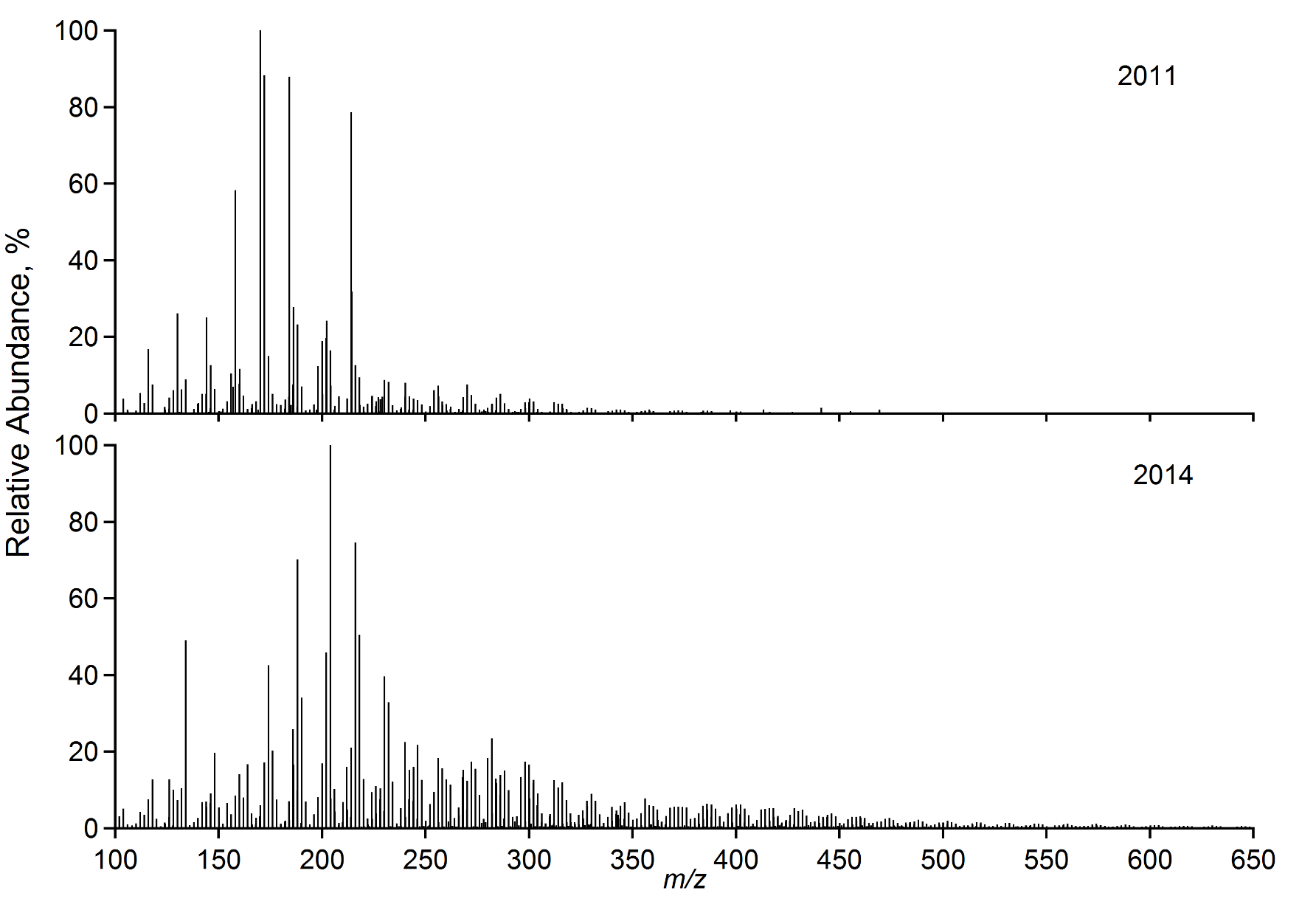
**

**Supplementary Figure S8.**

**
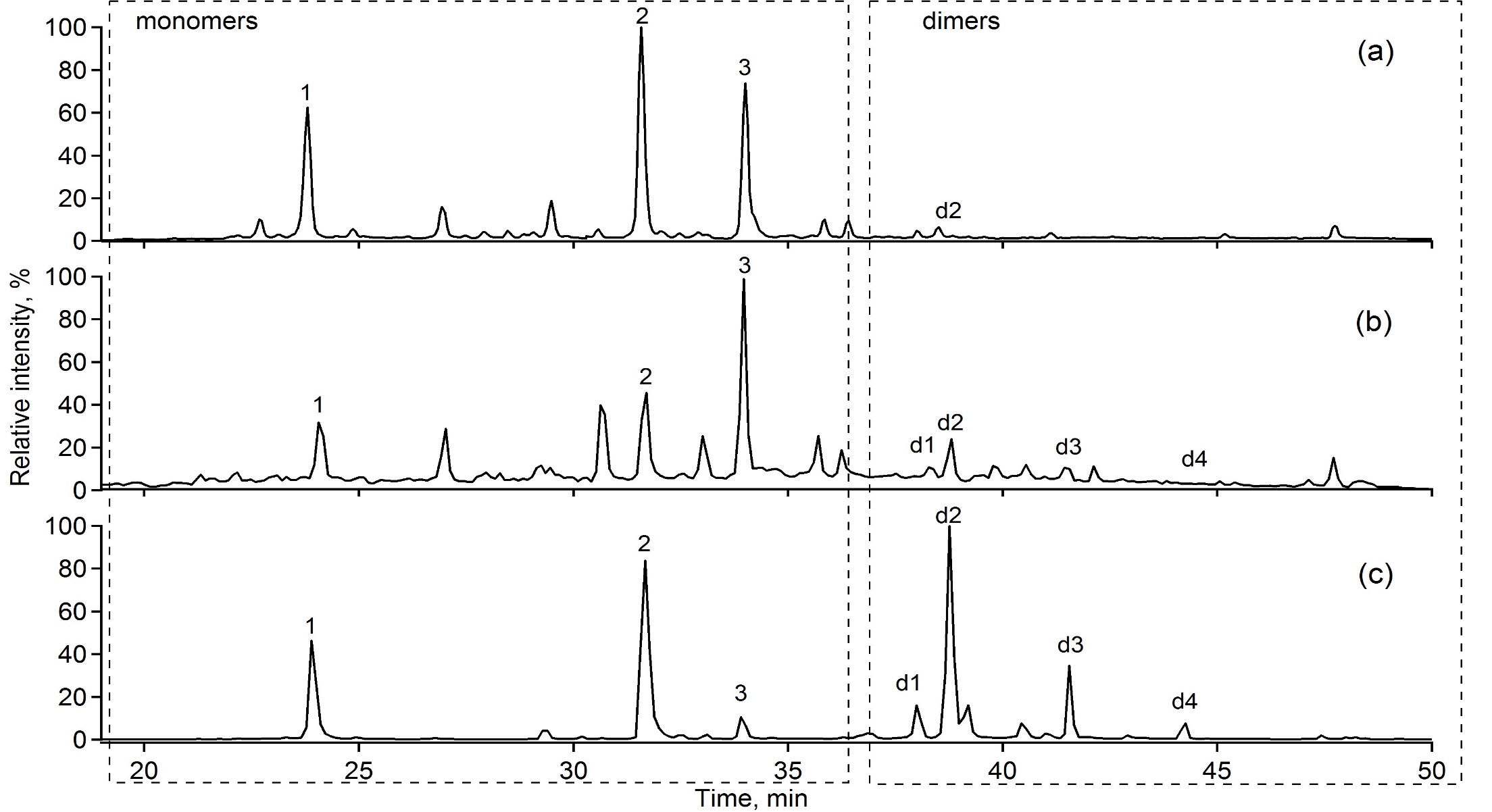
**

**Supplementary Figure S9.**

**Supplementary Figure S10.**

**Supplementary Figure S11.**

**Supplementary Figure S12**

**Supporting Information Figure Legends:**

**Supplementary Figure S1.** Monoterpene (a and b) and isoprene (c and d) concentrations during the sampling period in summer 2011 (blue markers) and 2014 (red markers) at Hyytiälä, Finland. While in 2014 monoterpene concentrations reached exceptionally high levels of up to 8 ppb, more typical concentrations below 1 ppb (with two short events reaching up to 2 ppb) were measured in 2011. Isoprene concentrations reached up to 1.5 ppb during summer 2014, while more typical concentrations below 0.25 ppb (with two short events reaching up to 0.5 ppb) were measured in 2011.

**Supplementary Figure S2.** Additional 15 masses observed with the PTRMS showing the decreasing gas phase yield as discussed in the main text (see Fig. 2e). With the exception of pinonaldehyde (i.e., the sum of the intensities of *m/z* 107, *m/z* 151 and *m/z* 169) and *m/z* 125 all masses show the same trend as discussed in Fig. 2e. Gas phase product yields are defined as concentration of oxidation product divided by the starting concentration of *α*-pinene.

**Supplementary Figure S3.** Direct infusion negative-nanoESI-Orbitrap mass spectra for SOA formed from ozonolysis of a mixture of *α*-pinene and limonene (56 ppb each) after long term ageing over 24 h (a) and 48 h (b) under natural day/night cycle aging. After 48 h of ageing, ions in the oligomeric region seem to be unaffected. Mass spectra of (c) *α*-pinene SOA (from 65 ppbv) and (d) of a BVOC mixture SOA (containing 65 ppbv of *α*-pinene and 60 ppbv of *β*-pinene, Δ_3_-carene and isoprene) ozonolysis experiments. While the distribution of oligomers changes in SOA from the BVOC mixture compared to the pure *α*-pinene SOA, the total number and molecular mass range are very similar in the two systems. Mass spectra of SOA from (e) *α*-pinene ozonolysis (with an OH scavenger present) and (f) OH radical oxidation conditions. Both oxidation regimes form an extensive number of dimers and trimers, although in distinctly different mass ranges. The figure (3e and 3f) was adapted from Ref. *8*.

**Supplementary Figure S4**. Ambient temperature (a and b), UV-B radiation (c and d) and PM_1_ concentration (e and f) during the 2011 (blue markers) and 2014 (red markers) sampling periods at Hyytiälä, Finland. Ambient temperatures reached almost 30 °C in 2014, whereas it was significantly cooler during the 2011 sampling period (maximum of 20 °C). In 2014, the maximum daytime UV-B radiation exceeded 2.5 W m^-2^ (except for two days) and reached up to 3 W m^-2^, whereas daily maxima during the 2011 sampling period were around 2 W m^-2^ or lower.

**Supplementary Figure S5.** (a) Oligomer fraction in individual samples (not ‘common ions’) as a function of organic carbon (OC) concentration during 2011 and 2014 sampling periods at Hyytiälä, Finland. The oligomer fraction is calculated as the sum of the peak intensities above *m/z* 280 to the total peak intensities over the entire mass range (*m/z* 100-650); (b) Oligomer fraction as a function of the monoterpene (left plot) and isoprene (right plot) concentrations, averaged over the sampling period of individual samples during the 2011 and 2014 campaigns at Hyytiälä, Finland.

**Supplementary Figure S6**. (a) Carbon oxidation state of Ozone-SOA and OH-SOA of all CHO compounds versus the number of carbons in a SOA compound. Each data point in the figure represents one or more compounds in the mass spectra with a distinct average oxidation state of its carbon atoms. Higher values indicate more oxidised compounds. OH-SOA components are clearly more oxidised than O_3_-SOA throughout the entire mass range; (b) Carbon oxidation state of all CHO compounds of ambient SOA in 2014 (common ions of 8 samples) and OH-SOA. OH-SOA is only slightly more oxidised than SOA in Hyytiälä from 2014 indicating a significant role of OH as oxidant for SOA formation in 2014; **(c)** Carbon oxidation state of all CHO compounds of ambient SOA in 2011 (common ions of 10 samples) and ozone-SOA. SOA from Hyytiälä from 2011 is slightly more oxidised than the pure ozone-SOA. The difference could be explained by a minor influence of OH oxidation; (d) Carbon oxidation state of all CHO compounds of ambient SOA in 2014 and 2011. SOA in 2014 is clearly more oxidized than in 2011 throughout the mass range.

**Supplementary Figure S7.** Combined 72-hour back trajectories for (a) the entire eight-day sampling period in summer 2011 clearly show the wide range of marine, natural terrestrial and anthropogenic source regions affecting the aerosol samples collected at Hyytiälä (marked with a cross on the maps) and 72-h back trajectories for two individual 12-h aerosol samples (b) 17-18 August, 2011 and (c) 22-23 August, 2011 showing distinctively different air mass histories. Warmer colours indicate a greater probability of a particle passing near the surface in a grid box. The maps were produced using IDL (Interactive Data Language) Version 8.2: <http://www.harrisgeospatial.com/ProductsandSolutions/GeospatialProducts/IDL.aspx>

**Supplementary Figure S8.** Direct infusion (−)nanoESI-Orbitrap mass spectra showing all components of a typical sample in 2011 (top) and in 2014 (bottom). The difference in the high-molecular weight mass region is clearly visible in the individual samples confirming different composition of the aerosol samples in these two years and the ‘common ion’ results presented in Fig. 1 in the main text.

**Supplementary Figure S9.** LC/(−)ESI-MS extracted ion chromatogram (EIC) for (a) pooled night-time ambient summer sample from Hyytiälä, Finland collected during 16 to 25 August 2011, (b) ambient summer sample from Hyytiälä collected during 25-28 July 2014, and (c) laboratory-generated sample from *α*-pinene/O_3_. The chromatographic peaks correspond to (1) diaterpenylic acid with *m/z* 171.0662, (2) *cis*-pinic acid with *m/z* 185.0818, (3) *cis*-caric acid with *m/z* 185.0818, (d1) dimer with *m/z* 377.1454, (d2) dimer with *m/z* 357.1552, (d3) dimer with *m/z* 367.1762 and (d4) dimer with *m/z* 337.1652. In the ambient sample (b) only one dimer (d2) was observed; the other small peaks between 36 and 42 min retention time in (a) do not correspond to d1, d3 and d4.

**Supplementary Figure S10.** Mixed boundary layer burden of cloud condensation nuclei (CCN) at 0.5 and 1% supersaturation as a function of temperature during 2011 and 2014 sampling periods at Hyytiälä, Finland. The median boundary layer burden of CCN increases with temperature and is noticeably higher during the warmer 2014 sampling campaign.

**Supplementary Figure S11.** Aerosol size distribution averaged over the filter sampling periods during the summer 2011 campaign at Hyytiälä, Finland.

**Supplementary Figure S12.** Aerosol size distribution averaged over the filter sampling periods during the summer 2014 campaign at Hyytiälä, Finland.
